# Supplementary material for: Optimizing CAR-T treatment: A T2EVOLVE guide to raw and starting material selection
Source: Mol Ther. 2024 Nov 12;33(3):847–65. doi: 10.1016/j.ymthe.2024.11.017 (PMC11897765; doi:10.1016/j.ymthe.2024.11.017)
Supplement: Document S1. Tables S1–S4 [file mmc1.pdf]

YMTHE, Volume *33*

Supplemental Information

Optimizing CAR-T treatment: A T^2EVOLVE guide to raw and starting material selection

Sergio Navarro, Carole Moukheiber, Susana Inogés Sancho, Marta Ruiz Guillén, Ascensión López-Díaz de Cerio, Carmen Sanges, Toshimitsu Tanaka, Sylvain Arnould, Javier Briones, Harry Dolstra, Michael Hudecek, Rashmi Choudhary, Inga Schapitz, Manel Juan, Nina Worel, Delphine Ammar, Maik Luu, Mirko Müller, Bernd Schroeder, Hélène Negre, and Paul Franz

Table S1: *Guidelines pertaining to the raw/starting materials used for CAR-T cell products manufacturing.*

Document type	Reference	Title	Topics	Note
**General ATMP guidance with chapters on raw/starting materials**				
EMA guideline	EMA/CAT/80183/2014 (replacing CPMP/BWP/ 3088/99)	**Guideline on the quality, non-clinical and clinical aspects of gene therapy medicinal products.**	Quality, non-clinical & clinical of ATMPs	**General guideline** presenting requirements for **gene therapy medicinal products,** for **MAA.** Raw/starting materials addressed in chapter 4 "Quality documentation".
EMA guideline	EMA/CAT/GTWP/671639/ 2008 Rev. 1	**Guideline on quality, non-clinical and clinical aspects of medicinal products containing genetically modified cells.**	Quality, non-clinical & clinical of ATMPs	**General guideline** presenting requirements for **products containing genetically modified cells**, for **MAA**. Raw/starting materials addressed in chapter 4 "Quality documentation".
EMA *draft* guideline	EMA/CAT/123573/2024 (previous reference: EMA/CAT/852602/2018)	**Guideline on quality, non-clinical and clinical requirements for investigational advanced therapy medicinal products in clinical trials.**	Quality, non-clinical & clinical of ATMPs	Guideline presenting requirements for **ATMPs** used in **clinical trials**. Raw/starting materials addressed in chapter 4 "Quality documentation".<br>*Draft guideline not adopted yet.*
European Commission guideline	C(2017) 7694	**Guidelines on Good Manufacturing Practice specific to Advanced Therapy Medicinal Products.**	Quality & GMP of ATMPs	**Guideline** on **GMP** for **ATMPs.** Raw/starting materials addressed in Chapter 7 ("Starting and raw materials"), Chapter 6 ("Documentation") and Chapter 2 ("Risk-based approach").
**Guidance dedicated to vectors**				
EMA reflection paper	CHMP/GTWP/587488/07	**Reflection paper on quality, non-clinical and clinical issues relating specifically to recombinant adeno-associated viral vectors.**	Quality, non-clinical & clinical topics	Reflection paper on the development of **recombinant AAV vectors**.
EMA guideline	CHMP/BWP/2458/03	**Guideline on development and manufacture of lentiviral vectors.**	Development & manufacture	Guideline dedicated to **lentiviral vectors.**
**Guidance applicable to biotechnology products and some vectors**				
ICH guideline	ICH Q5A Revision 2	**Viral safety evaluation of biotechnology products derived from cell lines of human or animal origin.**	Viral safety of biotechnology products (also applicable to certain vectors)	General guideline on viral safety of biotechnology products derived from cell lines of human or animal origin.<br>Following R2 revision (adopted in Nov 2023), guideline scope extended to become applicable to **certain viral vectors**, i.e., **only to viral vectors that are amenable to viral clearance steps.**
EMA guideline	CPMP/BWP/268/95	**The design, contribution and interpretation of studies validating the inactivation and removal of viruses.**	Viral safety of biotechnology products (also applicable to certain vectors)	Viral safety guideline on **the viral clearance studies (virus validation studies).** Guideline designed for biotechnology products, but **applicable to certain vectors (i.e., only to the vectors amenable to viral clearance steps).**

Guidance pertaining to materials of biological origin used to manufacture ATMPs				
European Pharmacopeia general chapter	Ph. Eur. 5.2.12.	**Raw materials of biological origin for the production of cell-based and gene therapy medicinal products.**	Quality of biological raw materials	Ph. Eur. chapter dedicated to **raw materials of biological origin** used for the production of **cell and gene therapy medicinal products.**
European Pharmacopeia general chapter	Ph. Eur. 5.1.7.	**Viral safety.**	Viral safety of biological materials	Ph. Eur. chapter dedicated to the **viral safety**, applicable to **materials of biological origin** used for the production of **cell and gene therapy medicinal products.**
European Pharmacopeia general chapter	Ph. Eur. 5.2.8.	**Minimising the risk of transmitting animal spongiform encephalopathy agents via human and veterinary medicinal products.**	Viral safety of biological materials	Ph. Eur. chapter dedicated to the **prion risk**, applicable to **materials of biological origin** used for the production of **ATMPs.**
European Pharmacopeia monograph	Ph. Eur. Monograph 2262	**Bovine serum monograph.**	Viral safety of biological materials	Ph. Eur. monograph dedicated to **bovine serum**.<br>Some parts of the monograph (such as viral testing) applicable to **bovine serum used as raw material** for the production of **ATMPs.**
European Pharmacopeia monograph	Ph. Eur. Monograph 0853	**Human plasma for fractionation monograph.**	Viral safety of biological materials	Ph. Eur. monograph dedicated to **human plasma** for fractionation. Some parts of the monograph (such as viral testing) applicable to **human serum derived products used as raw material** for the production of **ATMPs.**
EMA (CHMP/CAT) position paper	EMA/CHMP/BWP/353632 /2010	**CHMP/CAT position statement on Creutzfeldt-Jakob disease and advanced therapy medicinal products.**	Viral safety of biological materials	Position paper on the **prion risk** for **allogenic ATMPs**.<br>Applicable to **materials of biological origin** used for the production of **ATMPs.**
EMA guideline	EMA/CHMP/BWP/706271 /2010 (replacing CPMP/BWP/269/95)	**Note for guidance on plasma derived medicinal products.**	Viral safety of biological materials	Guideline dedicated to plasma-derived medicinal products. Principles for **collection of plasma-derived starting material** applicable to **allogenic leukapheresis** (used to manufacture **allogenic ATMPs**).
EMA guideline	EMEA/410/01	**Note for guidance on minimising the risk of transmitting animal spongiform encephalopathy agents via human and veterinary medicinal products.**	Viral safety of biological materials	Guideline applicable to **all ruminant-derived materials** used to manufacture medicinal products, including ATMPs.
EMA guideline	EMEA/22314/02	**Position Paper on re-establishment of working seeds and working cell banks using TSE compliant materials.**	Viral safety of biological materials	Position paper on the use of **ruminant-derived materials used for cell banks preparation.** Applicable to **recombinant vectors cell banks.**
EMA guideline	EMA/CHMP/BWP/457920 /2012 Rev 1 (replacing CPMP/BWP/1793/02)	**Guideline on the use of bovine serum in the manufacture of human biological medicinal products.**	Viral safety of biological materials	Guideline applicable to **all materials derived from bovine serum**, used to manufacture medicinal products.
EMA guideline	EMA/CHMP/BWP/814397 / 2011	**Guideline on the use of porcine trypsin used in the manufacture of human biological medicinal products.**	Viral safety of biological materials	Guideline applicable **to all materials derived from porcine trypsin**, used to manufacture medicinal products.

Table S2: *Different culture media used for CAR-T cell manufacturing.*

Type of Media	Name	Supplier
Serum-dependent media	RPMI-1640	Thermo Fisher Scientific
	AIM-V	Thermo Fisher Scientific
	CTS OpTimizer	Thermo Fisher Scientific
	IMDM	Thermo Fisher Scientific
Serum-free media or defined culture media	DMEM	Thermo Fisher Scientific
	mTESR	Stem Cell Technologies
	StemLine T cell expansion	SIGMA
	Prime XV cell expansion XSFM	Irvine Scientific
	Stem XV serum free human T cell base medium	R and D
	X-VIVO10	Lonza
	TexMACS	Miltenyi Biotec
	CellGenix T cell Medium	Cellgenix
	Prime-XV T cell CDM	Fujifilm
	LymphoONE T cell expansion Xenon-free Medium	Takara

Table S3: *Information required for a questionnaire for the selection of a raw material supplier.*

Area	Information requirements
**General Information**	- Contact details, number of staff, turnover, and organizational structure, as well as manufacturing sites (via Site Master File - SMF).<br>- It is recommended that a quality agreement or contract be established to clearly divide responsibilities between all parties involved.<br>- Access should be requested for auditing purposes to facilities, personnel, documents and pertinent financial information.<br>- Provision of information regarding the Quality Management System and compliance with regulatory requirements, including certification or accreditation level according to the ISO9000 family norm, GMP, or GLP is crucial.<br>- The availability of a Quality Manual or (Master) SOP for quality policy should be requested.
**Quality Assurance (QA) and Quality Control (QC)**	- Respectively, responsible personnel like Quality Managers.<br>- Questions about the system for Change Control and the management of Deviations.<br>- Data regarding inspections by authorities and/or other notified bodies if applicable.<br>- Information pertaining to Process Design and monitoring of problems, as well as the system for Corrective Actions and Preventive Actions (CAPA).
**Product and Process Innovation**	- Product and/or Process Innovation activity and provision of design improvements or innovations.
**Incoming materials**	- Tracing of incoming materials by lot or part number to the product, so the Chain of Identity (CoI) is guaranteed.<br>- Business continuity plans (or equivalent) that describe emergency measures taken in case of unexpected events.<br>- Provision of Certificate of Analysis (CoA) of all products.
**Quality Management Reports (QMR)**	- Concepts to drive continuous improvements and Quality Management Reports (QMR).

Table S4: *Additional information to consider before selecting a raw material supplier.*

**Expertise**	Current and future capabilities by scaling up to industrialization.
**Timing**	Subcontracting aspects, such as external QC, storage, and shipment.
**Stability**	The stability of the contract development and manufacturing organization (CDMO), including long-term viability and sustainability.
**Technology Transfer (TT)**	Possibility of transitioning to another CMO/provider due to mergers, acquisitions, or inability to scale up in terms of quantity and quality.
**Turnover of teams**	Ensuring continuity and expertise.
**Intellectual Property (IP)**	Protection and confidentiality measures.
**Cell bank** *if applicable*	Storage and maintenance.
**Costs**	Research and Development (R&D) compared to Good Manufacturing Practice (GMP) grade and considerations for change management.
**Devices used**	Equipment and technology employed in the manufacturing process.
