# Supplementary material for: Optimizing CAR-T treatment: A T2EVOLVE guide to raw and starting material selection
Source: Mol Ther. 2024 Nov 12;33(3):847–65. doi: 10.1016/j.ymthe.2024.11.017 (PMC11897765; doi:10.1016/j.ymthe.2024.11.017)
Supplement: Document S2. Article plus supplemental information [file mmc2.pdf]

Molecular Therapy
Review

Optimizing CAR-T treatment: A T^2EVOLVE guide to raw and starting material selection

Sergio Navarro,[1,14] Carole Moukheiber,[2,14] Susana Inogés Sancho,[3,14] Marta Ruiz Guillén,[3] Ascensión López-Díaz de Cerio,[3] Carmen Sanges,[4] Toshimitsu Tanaka,[5] Sylvain Arnould,[2] Javier Briones,[6] Harry Dolstra,[7] Michael Hudecek,[4,8] Rashmi Choudhary,[9] Inga Schapitz,[10] Manel Juan,[1] Nina Worel,[11] Delphine Ammar,[12] Maik Luu,[4] Mirko Müller,[8] Bernd Schroeder,[13] Hélène Negre,[2] and Paul Franz[8]

[1]Hospital Clínic de Barcelona, Secció d'Immunoterapia - Servei d'Immunologia, Barcelona, Spain; [2]Institut de Recherches Internationales Servier, Gif-sur-Yvette, France; [3]Clínica Universidad de Navarra, Hematología- Área de Terapia Celular, Pamplona, Spain; [4]Lehrstuhl für Zelluläre Immuntherapie, Medizinische Klinik und Poliklinik II, Universitätsklinikum Würzburg, Würzburg, Germany; [5]Astellas Pharma Inc, Tokyo, Japan; [6]Hospital de Sant Pau, Hematology Department, Barcelona, Spain; [7]Radboud University Medical Center, Department of Laboratory Medicine, Nijmegen, the Netherlands; [8]Fraunhofer Institute for Cell Therapy and Immunology, Leipzig, Germany; [9]Takeda Development Centers Americas, Inc, Lexington, MA, USA; [10]Bayer Vital GmbH, Leverkusen, Germany; [11]Medical University of Vienna, Department of Transfusion Medicine and Cell Therapy, Vienna, Austria; [12]Astellas Pharma B.V., Leiden, the Netherlands; [13]Miltenyi Biotec B.V. & Co KG, Bergisch Gladbach, Germany

Chimeric antigen receptor (CAR)-T cell products, classified as Advanced Therapy Medicinal Products (ATMPs), have shown promising outcomes in cancer immunotherapy. The quality of raw and starting materials used in manufacturing is critical to ensure the efficacy and safety of CAR-T cell products and depends primarily on the selection of the right materials and the right suppliers. It is essential to consider a long-term strategy when selecting raw and starting materials to prevent delays in the supply of innovative, high-quality, and safe therapies to patients. A thorough assessment will allow developers not only to select suppliers who comply with regulatory requirements but also to ensure a sustainable supply of materials throughout the development and the commercial phases. A careful selection of materials and suppliers can avoid the need of comparability studies due to changes in the supply of materials, impacting costs and causing significant delays in development and treatment readiness for patients. This work, coordinated by the T^2EVOLVE IMI consortium, provides guidance for the selection and handling of raw and starting materials. By following these suggestions, developers can ensure that they use high quality raw and starting materials through the product development and life cycle, resulting in safe and effective CAR-T therapies for patients.

INTRODUCTION

CAR-T cell products have emerged as a promising approach to treat a wide range of cancers including hematological malignancies. However, the development and manufacture of these ATMPs involve complex processes that require strict adherence to regulatory guidelines and quality control measures. One of the critical factors influencing the safety and efficacy of these therapies is the use of high-quality good manufacturing practice (GMP) raw and starting materials.

The definition of starting materials is provided in Part IV of the Annex to Directive 2001/83/EC on the community code relating to medicinal products for human use. A starting material is any substance used in manufacturing that is incorporated as a significant fragment into the structure of the active substance. For CAR-T cell products, starting materials include entities such as viral vectors, transposons, genome editing components, plasmids used for viral vector production, and cellular source materials such as leukapheresis products.

The definition of raw materials is also provided in Part IV of the Annex to Directive 2001/83/EC on the community code relating to medicinal products for human use. Raw materials (also called ancillary materials in the United States) are substances used in the manufacture or extraction of the active substance(s), but from which the active substance is not directly derived, such as reagents, culture media, cytokines, additives, and buffers used in chromatography, and so on (Figure 1).[1]

Importance of the quality of starting and raw materials

The quality of starting and raw materials is crucial for the development of CAR-T cell products. According to Ph. Eur. 5.2.12, it is the responsibility of the user of a raw material to ensure that it is of suitable quality for the intended use.[2] The raw materials used in the production of CAR-T cell products can have a critical impact on the

https://doi.org/10.1016/j.ymthe.2024.11.017.

[14]These authors contributed equally

Correspondence: Sergio Navarro, Hospital Clínic de Barcelona, Secció d'Immunoterapia - Servei d'Immunologia, Barcelona, Spain.
E-mail: senavarro@clinic.cat
Correspondence: Carole Moukheiber, Institut de Recherches Internationales Servier, Gif-sur-Yvette, France.
E-mail: carole.moukheiber@servier.com
Correspondence: Susana Inogés Sancho, Clínica Universidad de Navarra, Hematología- Área de Terapia Celular, Pamplona, Spain.
E-mail: sinoges@unav.es
Correspondence: Paul Franz, Fraunhofer Institute for Cell Therapy and Immunology, Leipzig, Germany.
E-mail: paul.franz@izi.fraunhofer.de

© 2024 The Authors.
Published by Elsevier Inc. on behalf of The American Society of Gene and Cell Therapy.
This is an open access article under the CC BY license (http://creativecommons.org/licenses/by/4.0/).

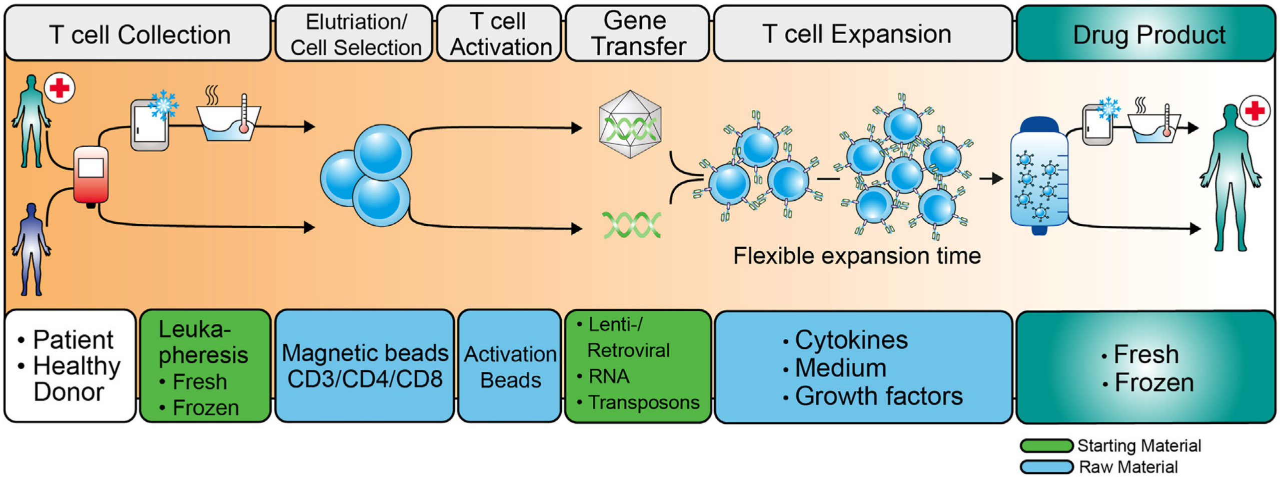

Figure 1. Starting material and raw material options for CAR-T cell products manufacturing processes
The manufacturing processes for CAR-T cell products are divided into distinct phases, each of which requires a specific combination of raw materials (blue) and starting materials (green). The selection of these materials is of crucial importance with regard to the quality of the final CAR-T cell product.

quality, safety and efficacy of the final product, thereby affecting patient safety.

When a developer of an innovative CAR-T cell product reaches the point of conducting Good Laboratory Practice toxicology studies in animals, the critical starting and raw materials must be established, as the final product used in the toxicology studies must be representative of the final product used in phase 1 clinical trials and later in phase 2/3 clinical trials.

The quality of the starting and raw materials may be considered according to the stage of development of the CAR-T cell product, thereby acknowledging the inherent evolution of the quality profile of the medicinal product during its pharmaceutical and clinical development. Nevertheless, patient safety needs to be ensured in early phase clinical development. The aim is to have an appropriate qualification strategy for these materials when used for the manufacture of CAR-T cell products. It should be noted that changes in starting or raw materials during the life cycle of the CAR-T cell product may affect the quality of the final product and thus require additional studies to demonstrate comparability. The impact of this material on the quality, safety, and efficacy of the CAR-T cell product has to be evaluated using a risk-based approach.

Sources of information on starting and raw materials

Apart from general GMP guidelines, various literature is available regarding the quality and selection of starting and raw materials.[3–5] One possible source of more in-depth information is the International Pharmaceutical Regulators Program (IPRP), of which the European Medicines Agency (EMA) is a member. The IPRP regularly publishes comments or reflection papers, for example, on general considerations for raw and starting materials used in the manufacture of cell and gene therapy products.[6] These include considerations of risks posed by raw and starting materials used in the manufacture of ATMPs and recommendations for risk management, with general guidance on an appropriate quality management system or tests and assays for raw material qualification. Often, however, these considerations simply recall or refer to official guidelines, such as ICH Q9 (Quality Risk Management),[7] without sharing specific procedures or real-world experience.

A second source of information on starting and raw materials for production of CAR-T cell products are commentaries or review articles, where authors provide, for example, general insights into the two-step raw material qualification process and detailed information on establishing an action plan and priorities based on failure mode effect analysis risk assessment.[8] Examples of EU requirements for registration of production materials and best practice for effective control are also provided.[4,9]

A third way to gain insight into the starting and raw material selection process is to obtain white papers or e-books directly from suppliers, focusing mainly on their own products and services, such as expansion media. Highlighted also are various parameters that should be considered in terms of their impact on the quality of the final product, and often aspects, such as secure supply chain, global compliance, regulatory support, that should be considered in terms of supplier.[10]

What is consistently missing from these available documents are specific procedures on how to prepare starting and raw materials, how to implement measures to ensure viral safety, and how to identify the right suppliers for sourcing high quality materials—important steps in establishing the manufacturing process of CAR-T cell products.

Viral safety of CAR-T cell products

The viral safety of starting and raw materials is a key parameter to be taken into account in the development of CAR-T cell products, to

reduce risks for transmission of infections by conventional viruses or prions (i.e., agent causing transmissible spongiform encephalopathies [TSE] and variant Creutzfeldt-Jakob disease).

Meticulous selection of all starting and raw materials used, is needed to reduce the risks of introducing viral contaminants in the manufacturing process of CAR-T cell products.

All the starting and raw materials of biological origin used in the CAR-T cell product manufacturing process have to be taken into consideration, including the following:

(1) Peripheral blood mononuclear cells (PBMCs) used as starting material (leukapheresis product).
(2) Vectors.
(3) Serum-derived products.
(4) Proteins produced by recombinant DNA technology, such as growth factors, cytokines, hormones, enzymes, and monoclonal antibodies (conjugated or non-conjugated).
(5) Proteins extracted from biological materials, such as animal-derived enzymes, polyclonal antibodies, and proteins/peptides of biological origin (e.g., albumin, transferrin).

The viral safety of all the raw and starting materials used is crucial to ensure the safety of CAR-T cell products for patients, even in early phase clinical trials.

Aim

As raw and starting materials are crucial for the quality of GMP manufactured CAR-T cell products and, therefore, also for patient safety, the aim of this article is to summarize all important aspects of the careful selection of these materials. The importance of the composition of autologous starting material from patients with malignancies and how washing, formulation, and cryopreservation of the leukapheresis product after collection can affect cell quantity and quality. Additionally, how the presence of certain cell subsets during elutriation/cell purification and culture initiation can negatively affect purity, T cell activation and expansion, will be addressed.[11] In contrast, the requirements for the procurement of allogeneic T cells are also highlighted.[12] Finally, regulatory aspects regarding the selection of starting and raw material are also discussed.

STARTING MATERIALS FOR CAR-T CELL PRODUCT MANUFACTURING

This section focuses on the two key starting materials for CAR-T cell products: the leukapheresis product and the viral or non-viral vectors encoding the CAR.

Leukapheresis product

CAR-T cell products are manufactured using non-mobilized PBMCs collected by leukapheresis.[13] Although autologous cells are currently used in all approved CAR-T cell products and in the vast majority of clinical trials, preparation from allogeneic leukocytes is an interesting strategy to consider.[14]

Indeed, patients with malignancies often have lower leukocyte, CD3, and CD4 counts; an inverse CD4/CD8 ratio; and more activated lymphocytes. In particular, low target cell counts can negatively affect the purity of the leukapheresis material and collection efficiency. In leukemic diseases, a high proportion of blasts may also be critical for the quality of the collected PBMCs.[15] Furthermore, patient pretreatment can have a negative impact on the immune system, and many anticancer drugs cause reversible T cell dysfunction, which can be mitigated by observing certain washout periods.[16]

Allogeneic CAR-T cell products use PBMCs collected by leukapheresis from untreated healthy donors, which have high numbers of compatible PBMCs and provide a more uniform starting material.[12,17] As a result, the cells show a faster growth behavior and metabolic fitness, which has a positive impact on the manufacturing process and results in a better final product to be infused.

The relevant guidelines applicable to leukapheresis products are listed in Table S1.

Quality control on the leukapheresis product

Quality control of leukapheresis has two main objectives: the quality of the leukapheresis product as a starting material, and its safety (microbial and viral).

Quality of the leukapheresis. Once the leukapheresis is collected, the material is tested to certify the amount and characterize the populations of different cells and contaminants. It is crucial to perform at least two tests to ensure these parameters: hemogram (at least leukocytes, differential counts, and hematocrit counting) carried out by the collection facility, and T cell, B cell, and natural killer cell subpopulations profiling carried out by the manufacturer, to calculate the number of T cells, to start production.

In addition, microbial testing is essential to ensure the sterility of the starting material. In the majority of cases, microbial contaminations derive from the patient and are mainly related to bloodstream infections or contaminated peripheral or central venous lines. Nevertheless, the potential for microbial contamination of the leukapheresis product remains a concern, particularly in instances where tubing sets and bags used in the procedure exhibit signs of leakage or breakage.

Responsibility for microbiological testing of the leukapheresis product ultimately lies with either the collection facility or the CAR-T cell manufacturing facility, depending on the regulatory requirements in each EU member state. Based on the authors' experience, several cases illustrate this variability. One option for CAR-T cell manufacturers would be to sample the leukapheresis product before manufacturing and only conduct testing if microbial contamination is detected in the final product. However, there are also more cautious procedures, for example at the Hospital Clinic of Barcelona, where the patient's blood is initially tested for various markers of infection before leukapheresis. In the event that the apheresis product has been tested and

Table 1. Measures to ensure the viral safety of an allogeneic leukapheresis

Aim	Principle	Detailed viral safety requirements
1. Selection of donors and exclusion criteria	The selection and exclusion criteria of donors shall comply with EU and US guidelines and directives.<br>The donors' selection includes the donors' interview and screening for markers of infection.	Donors' questionnaire and interview: questionnaire on medical history (including past illnesses, recent infections, treatments, history of corneal or dura matter graft, history of treatment with medicinal products derived from human pituitary glands (i.e., growth hormones), history of treatment with blood products), recent traveling, family history predisposing to TSE, history of injectable drug use, donor's lifestyle (e.g., piercing, tattoos, sexual orientation)
		Donor's screening for markers of infection with known viruses (including HIV, hepatitis C virus, hepatitis B virus, CMV, WNV).
		For medicinal products to be marketed in Europe, only "voluntary and unpaid blood donations" should be used.
2. Testing of donors and/or donations for markers of infection with known viruses	Testing for human viruses and infectious diseases should be performed in compliance with EU and US regulations.<br>Testing plan to be adapted according to local requirements.	Testing for viruses and infectious diseases should include (but not limited to) HIV-1/2, HCV, HBC, HAV. It can also include EBV, CMV, HTLV-I/II, WNV and any other emerging infectious viruses (based on the country and date of collection) (i.e., Ebola virus, Zika virus).<br>Note: the tests should be performed with marketed testing kits. If "in-house" assays are used, validation data should be available.
3. Traceability	A traceability system has to be in place which enables each donation to be traced from the donor through to the finished medicinal product and vice versa, to ensure that the manufacturer would be informed if, in exceptional circumstances, post-collection information would lead to measures regarding the product.	–
4. Post-collection measures	A post-collection information system should be in place including measures for reporting of serious adverse reactions which may be caused by the quality or safety of the blood-derived starting material.	–

found to be negative, it is then sent to the manufacturing unit, where a sample is taken for analysis to ascertain whether there is any microbial contamination, for example, in the bag or picked up during the leukapheresis procedure. Following the manufacturing process, samples are taken from the final CAR-T cell product for release-relevant microbiological controls (e.g., BacTec, qPCR, etc.). A comparable procedure is conducted at the Department of Haematology and Cell Therapy at the University of Navarra. Before leukapheresis, patients are tested for compliance with microbiological criteria. Subsequently, the apheresis material is subjected to testing at the manufacturing facility to ascertain its suitability as a starting material. Also, at the Department of Transfusion Medicine and Cell Therapy, Medical University of Vienna, all patients undergoing the collection of starting material and all final CAR-T cell products are subjected to microbial testing. In certain instances, leukapheresis products are transported in a fresh state to the manufacturing authorization holder (MAH). These starting materials are then subjected to microbial sterility testing, the results of which are typically available within 1–2 days. Some manufacturers (e.g., Kite Pharma) will proceed with CAR-T cell manufacturing even if the leukapheresis product is tested positive, due to the use of antibiotics in the culture medium and multiple washing steps, which greatly reduces the likelihood of contamination. In contrast, other MAHs (e.g., Novartis) would refrain from proceeding with the manufacturing process in the event of being informed of a contaminated leukapheresis product.

Once the required number of T cells is obtained, the leukapheresis material is ready to be used as the starting material for manufacturing CAR-T cell products.

Viral safety of the leukapheresis. Depending on whether leukapheresis is autologous or allogeneic, the requirements to ensure viral safety will be slightly different.

In the case of allogeneic CAR-T cell products, the starting material is leukapheresis from healthy donors who have been carefully evaluated and adequately tested for infectious transmissible agents. These materials must comply with appropriate EU and/or national legislation applicable to transplantation and transfusion.

The viral safety of the allogenic leukapheresis is ensured by a combination of measures. Table 1 provides an overview of the complementary measures taken to ensure the viral safety of the allogeneic leukapheresis. For detailed information on the requirements, please refer to EU

Table 2. Advantages and disadvantages of using fresh versus cryopreserved leukapheresis

			Reference
Fresh leukapheresis	Disadvantages	Patient access limited to a specific geographical region	Tyagarajan et al.[18]
		Restricted time available to start manufacturing (limited window of time: 24–48 h)	
		No flexibility allowed in scheduling for shipping	
		No flexibility allowed depending on patient health status	
		Need to have manufacturing slots reserved for a given patient	
		Limited time to perform quality control testing on the leukapheresis product before use (especially sterility)	
	Advantages	Optimal cell viability	
Cryopreserved leukapheresis	Disadvantages	Need for storage space for cryopreserved leukapheresis material	
		Need for standardization of cryopreservation/thawing procedures	
		Need for specialized equipment and trained personnel	Meneghel et al.[19]
		Risk of freeze-thaw losses in viability and recovery for cryopreserved leukapheresis material	Tyagarajan et al.[18]
	Advantages	Flexibility in scheduling leukapheresis: Collection of cells before applying appropriate bridging therapy without a risk to T cells (also possible for fresh products, unless there is no long distance transport)	
		Protection from external factors (e.g., shipping delays due to extreme weather conditions)	
		No manufacturing time constraints to start the manufacturing campaign	
		Elimination of platelets and red blood cells post thawing	
		Use as backup leukapheresis material to repeat manufacturing in case of production failure or for patients re-dosing	Elavia[20]
		Time to perform quality control testing on leukapheresis product	Wang and Rivière[21]
		Possibility to process the leukapheresis material in different sites regardless of the geographic location of the manufacturing sites vs. the leukapheresis collection	Xu et al.[22]

and/or US guidelines and directives and/or any local additional testing requirements for tissues and cell donors.

In the case of autologous CAR-T cell products, where leukapheresis material obtained from the individual patient is used as a starting material, the viral safety requirements for the leukapheresis are simplified and include the following:

(1) Screening of each patient for viral and bacterial infectious diseases (including at least HIV-1/2, hepatitis C virus, hepatitis B virus, and syphilis) to be performed before collection of cells for manufacturing, according to EU and/or US guidelines and directives, and/or any local additional testing requirements for tissues and cell donors. Leukapheresis may be postponed in case of an active viral infection.
(2) Traceability system, enabling the identification of a patient's starting apheresis material through to the final product.

Fresh versus frozen leukapheresis as the starting material

The leukapheresis material can be used either fresh or after cryopreservation. Currently, the majority of commercial CAR-T cell products are manufactured from fresh leukapheresis products packed in their own specified shipping containers (2°C–8°C). In contrast, Kymriah/Tisagenlecleucel (Novartis) starts from a cryopreserved leukapheresis product.

Overall, the main reasons for starting a manufacturing process for CAR-T cell products from frozen leukapheresis material instead of fresh leukapheresis material include the following:

(1) The CAR-T product is approved using frozen leukapheresis material.
(2) To avoid the risk of leukapheresis material expiring in the event of transport delays, long distance transport to the manufacturing site, or high occupancy of the GMP facility.
(3) Storage of frozen backup leukapheresis material for re-manufacturing.

Table 2 provides an overview of the advantages and disadvantages of fresh and frozen leukapheresis products. The section below gives a concrete example of a stability study supporting the use of fresh apheresis for the CD19 CAR-T cell clinical trial ARI0001 phase 1 (NCT03144583).

Stability study on leukapheresis product: Experiences from the Hospital Clinic of Barcelona

The immunotherapy team of Immunology Service from Hospital Clinic of Barcelona performed a stability study on fresh leukapheresis up to 48 h, in which a cell viability of more than 95% over the course of the 2 days could be demonstrated. However, it is not always possible to start the manufacturing of the CAR-T cells in time

Table 3. Parameters analyzed in CAR-T cell products manufactured for ARI0001 phase 1 study (NCT03144583)

Variable	Average		Standard deviation		P(T < t)
	Fresh	Frozen	Fresh	Frozen	
Coefficient of expansion	25.77	14.61	11.15	17.03	0.051
Population doubling level	4.53	3.35	0.74	1.15	0.004
Doubling time (h)	43.98	67.71	11.03	20.84	0.002
Growth rate (cells/day)	0.392	0.264	0.061	0.068	2×10^{-5}
Transduction efficiency (%CAR)	36.90	29.47	1.42	1.28	0.103

and is thus necessary to cryopreserve the leukapheresis material. Therefore, it is essential to validate that it is possible to manufacture CAR-T cells from cryopreserved leukapheresis starting material.

Different parameters from beginning to end of the manufacturing process were studied. Based on the results, it was concluded that the production from fresh and frozen leukapheresis products is almost the same. Specifically, the following parameters were studied in products manufactured for ARI0001 phase 1 study (NCT03144583).

(1) Coefficient of expansion (final cell count/initial cell count).
(2) Population doubling level (number of doublings succeeded).
(3) Doubling time (hours).
(4) Growth rate (cells/day).
(5) Efficiency of transduction (%CAR).

Results showed that the cells from frozen leukapheresis material grew slower, but the final amount and percentage of CAR-T cells were approximately equal to those from fresh leukapheresis (Table 3, no significant differences observed). Furthermore, the desired doses for patient treatment were achieved in all cases (target dose 1×10^6 CART+/kg, depending on patient's weight).

For that reason, the study concluded that the best option when using frozen leukapheresis is to start the culture of CAR-T cells by doubling the quantity of T cells, to achieve a similar number of CAR-T cells at the end of the manufacturing process.

Conclusion:

(1) Fresh production can start up to 48 h after the end of leukapheresis.
(2) CAR-T cell products manufactured by both strategies were acceptable as both yield the required final doses.
(3) CAR-T cells manufactured from frozen leukapheresis grow slower and take longer to initiate replication cycles.
(4) CAR-T cells manufactured from both strategies have similar transduction levels and clinical response times.
(5) Both fresh and frozen leukapheresis strategies are valid for the manufacturing of CAR-T cell products; however, cryopreservation of leukapheresis material allows for a more efficient organization of manufacturing.

Viral vector

Production and control of viral vectors

There are different types of viral vectors when transducing CAR molecules into T cells to convert them into CAR-T cells. Currently, the two most commonly used viral vectors are recombinant lentiviral and retroviral vectors. Additional viral vector types are also used, for example recombinant adenoviral and associated adenoviral vectors. Recombinant-associated viral vectors are generally used as additional vectors to carry out gene editing or gene modification (insertion or deletion). In recent years, the lentiviral vectors have become more widely used.[5,21]

The choice of vector type depends on the manufacturing process used and also on the intended purpose of the vector transfer. If a single gene or multiple genes are to be transduced, there is a choice of using one or two vectors (recombinant lentiviral vector [rLV] vs. rLV + recombinant adeno-associate virus). The size of the expression cassette delivered by the vector has a direct impact on the yield of each batch, while each viral vector has its specific loading limit. Choosing the level of expression (constitutive, inducible, or locus dependent) and the cell to be targeted will affect the choice of the viral vector. Finally, during process development, the choice of the right viral vector should be considered to (i) reduce the cost of goods, (ii) achieve a high transduction efficiency, and (iii) obtain the most viable and potent CAR-T cells to be administered to the patient.

Main steps of lentiviral vector production: Experiences from the Hospital Clinic of Barcelona

The manufacturing of a CAR lentiviral vector is carried out in a clean room (GMP, negative pressure). The production of each batch of viral supernatant (4L) takes approximately 12 days per batch, using the HEK293T packaging cell line and 1L culture flasks, in addition to polyethyleneimine. Subsequent concentration of virus at 40× is performed by tangential flow filtration, in addition to PBS diafiltration (Figure 2). Ten milliliters of lentiviral aliquots are then prepared, and stored at −80°C until use.

The lentiviral vector is produced using a cell bank from a cell line of human origin (HEK293). For each production, HEK293T cell banks are first expanded in T175 flasks for two passages. Cells are then transferred to four 10-layer CellStacks cell culture chambers (Corning) and one 1-layer CellStack to control cell proliferation. Plasmid transfection is carried out the next day using 3.86 mg polyethyleneimine, 763 mg transfer vector, 377 mg pMDLg-pRRE, 188 mg pRSV-Rev, and 221 mg pMD2.G per liter. Viral supernatants are collected 2 days later and clarified using a 0.45-mm polyvinylidene fluoride membrane. Four liters of viral supernatant are concentrated and diafiltered using KrosFlo Research IIi Tangential Flow Filtration System (Spectrum Labs) and 500 kD modified polyethersulfone hollow fibers. Two liters of PBS is used as diafiltration buffer.[23] For release of the lentiviral vector product, quality control tests are performed at the

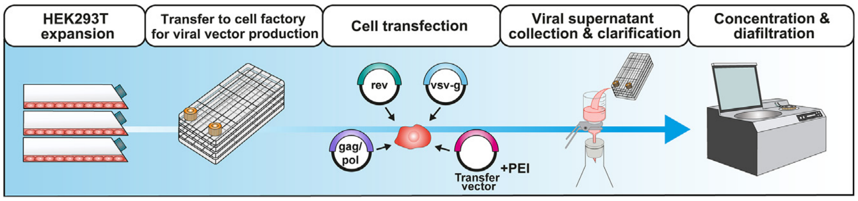

Figure 2. Viral vector production at the Advanced Therapies Unit of the Immunotherapy Section of Immunology Service of the Hospital Clinic of Barcelona

The production of lentiviral vectors carrying the expression cassette encoding an anti-CD19 CAR is initiated with the expansion and transfer of HEK293T cells (left), which are subsequently transfected with the corresponding plasmids by the addition of polyethyleneimine (PEI). Subsequently, the cell supernatant is collected and clarified, and the viral vector suspension is concentrated using tangential flow filtration and diafiltration (right).

Hospital Clinic of Barcelona to assess the correct quality attributes of the lentiviral vector (Table 4).

The manufacturing of the plasmids and lentiviral vectors must be performed under GMP conditions. At the end of vector production, each batch is tested for replicability on a reference cell line to assess the risk of infection by the vector. In addition, at the end of CAR-T cell manufacturing, replicability is re-assessed at one of the last manufacturing steps on product manufactured in the last culture media used. The most common method used for this purpose is PCR. Genes like Env are detected in DNA samples extracted from CAR-T cell DNA. If no bands are detected in the PCR amplification gel, replication-competent lentiviruses are absent from the CAR-T cell product. To avoid a false positive, it is recommended to select or request from a testing company the use of a specific primer not able to amplify any residual plasmid for vector manufacturing, as some primers could target common sequences between the vector and plasmids used to manufacture the vector.

The relevant guidelines applicable to vectors are listed in Table S1.

Viral safety of viral vectors

Vectors are biotechnology products prepared using a cell line of human or animal origin with materials of biological origin used in the manufacturing process.

The viral safety of vectors can be achieved using a combination of the following measures:

(1) Careful selection of the cell line and materials used in the vector manufacturing process: All the materials of biological origin used to manufacture the vector must be identified (including materials used for cell line establishment, cell banking, and production), and viral safety data must be available for each material of biological origin, in regard to conventional viruses and prions (TSE agent).
(2) Testing for the presence of viral contaminants performed at different stages of the vector production, in particular on the vector cell banks, process intermediates, and/or on each vector batch.
(3) Implementing viral clearance steps (if possible) in the vector manufacturing process (i.e., effective steps to remove or inactivate potential viral contaminants), and performing viral clearance studies (virus validation studies) for viral clearance steps (if applicable). Indeed, the vector manufacturing process depends on the type of vector:

- Some vector types are amenable to viral clearance (which means that their manufacturing process can include effective steps for viral inactivation/removal), without a negative effect on the vector. The viral safety of these vector types amenable to viral clearance measures must comply with ICH Q5A (R2) guidelines.
- Other vector types are not amenable to viral clearance, due to a resulting negative effect on the vector. This means that viral inactivation/removal steps cannot be included in their manufacturing process. Therefore, the viral safety of these vector types will mainly rely on the other two measures (i.e., selection of materials and viral testing).

Non-viral vectors

CAR-T cell products modified with gamma-retroviral or lentiviral vectors to express a CAR have become standard in immunotherapy and show remarkable efficacy in clinical trials and commercial pharmaceutical use. However, as the associated costs and regulatory requirements, as well as safety concerns, are barriers to rapid and widespread clinical translation of novel T cell-related therapies, alternative transfection technologies have been explored.[24] These methods can allow either sustained or transient expression of the CAR construct.

Sleeping beauty transposon system

The Sleeping Beauty (SB) transposon system is an interesting method for transferring DNA that combines permanent genomic insertions with simple and inexpensive manufacturing (in contrast with viral vectors).[25] It includes a class II transposon and a transposase enzyme to cut and paste donor DNA that has SB-specific inverted repeats/direct repeats into chromosomal DNA. This results in highly efficient transfer of desired genetic material into the genomes of host cells of vertebrate species.[26] This feature uniquely positions transposons as non-viral gene delivery systems capable of efficient genomic integration that can be used as tools for versatile applications in genetic engineering, including gene therapy. This system has been used to generate CAR-T cells and entered the clinical stage with two clinical trials adopting CD19 CAR-T cell products for targeting minimal residual disease of

Table 4. Quality controls (QCs) performed at the Hospital Clinic of Barcelona of large-scale lentiviral production for release of the lentiviral vector

Parameter	Acceptance criteria	Method
Appearance	yellowish aqueous solution	visual observation
pH	6.9–7.8	Ph. Eur. 2.2.3
Infectious particles	$\geq 3.75 \times 10^7$ particles/mL	limiting dilution
p24	$\geq 3.75 \times 10^5$ pg/mL	ELISA
Ratio infectious particles/p24	$\geq$7 TU/pg	calculation
Potency	$\geq$1.17	co-culture/flow cytometry
Identity	identity confirmed	PCR/sequencing
Sterility	no growth	Ph. Eur. 2.6.27
Endotoxins	$\leq$4 EU/mL	Ph. Eur. 2.6.14
Mycoplasma	absent	Ph. Eur. 2.6.7
Adventitious virus	absent	qPCR
Residual host DNA (HEK293)	$\leq$50 ng/mL	qPCR
Residual plasmid	$\leq$10 ng/mL	qPCR
Residual host protein (HEK293)	$\leq$50 μg/mL	ELISA
Residual BSA	$\leq$1 mg/mL	ELISA
Aggregates	$\leq$10%	nanoparticle tracking analysis
Replication-competent lentivirus	not detectable	qPCR

non-Hodgkin lymphoma and acute lymphoblastic leukemia after hematopoietic stem cell transplantation in 2011.[27] In recent years, there have been significant advancements in SB technology that were crucial to overcoming issues of unstable genomic transfer and low cell viability arising from the use of naked plasmid DNA.[28] It has been demonstrated that CAR-T cells can also be generated by SB transposition of CAR genes using minimalist DNA vectors called minicircles (MCs) and either mRNA or recombinant protein to encode the transposase.[29] CD19 CAR-T cell products generated via the enhanced SB approach exhibited potent reactivity *in vitro* and eradicated lymphoma in a xenograft model *in vivo*.[30] Interestingly, electroporation of SB MCs is significantly more efficient and less toxic compared with conventional plasmids, enabling the cost-effective, rapid production of therapeutic doses of CAR-T cell products.[24,31] This approach has led to the first-in-human clinical trial with SLAMF7 CAR-T cells to treat multiple myeloma, the CARAMBA study.[32]

PiggyBac transposon system

The PiggyBac (PB) system is also a class II transposon that was originally isolated from the cabbage looper moth *Trichoplusia ni* genome.[33] The PB transposase mediates exchange of genetic materials between the vector and host genome through recognition of inverted terminal repeat (ITR) sequences present on the transposon vector and the corresponding TTAA sequences present in the genome, a cut-and-paste mechanism to move DNA, like the SB system. Transgenes flanked by the ITRs can, therefore, be integrated stably into TTAA integration sites. A first study in human cells for evaluating PB as alternative gene transfer method for human genome demonstrated a lack of overproduction inhibition and precise excision.[34] Additionally, in contrast with SB, the PB system has a larger cargo size allowing for the insertion of larger genes or multiple transgenes simultaneously.[35] Different optimizations of the system have been performed in past years to expand PB application in cell and gene therapy and allow sustained genetic modification of T cells.[36,37] Encouraging preclinical evidence[38,39] and optimization of the manufacturing protocols (9) allowed clinical applications of the PB mediated CAR-T cell products for treatment of both hematological and solid tumors.[40–42]

CAR-mRNA-modified T cells

Compared with integrating DNA transfer technologies, transferring mRNA via electroporation or cationic lipid-mediated transfection generates T cells with a transient CAR expression for a few days, as has been studied and reported.[43,44] This approach has been found to produce antitumor reactivity for a limited time, offering potential safety benefits that could result in lower toxicity in hematological and solid tumor settings. Despite this, very few clinical trials have been registered using RNA-modified CAR-T cells,[45,46] and only a few protocols for clinical grade, GMP-compliant, mRNA-based CAR-T cell manufacturing are available.[47] Interestingly, the transfection technology utilized is defined electroporation (EP) technology, whereas lipid nanoparticles seem to be an interesting alternative to overcome the membrane damage caused by the electric field applied on cells, as demonstrated in a recent comparative study.[48]

Viral and non-viral vector technologies are complementary in the CAR-T manufacturing vector toolbox. The limitations of one (e.g., multiple vs. single expression) can be successfully tackled by a second one. As CAR-T cell products become more complex with several functions associated or presented by the CAR-T cell products (single or multiple CART expression, interleukin (IL) expression, gene silencing), multiple technologies may be required within the CAR-T cell product manufacturing platform.

RAW MATERIALS FOR CAR-T CELL PRODUCT MANUFACTURING

The quality of starting and raw materials used in the production of CAR-T cell products plays a pivotal role in determining the therapeutic success and safety of the medicinal product. Despite their importance in the production of ATMPs, raw materials are only regulated according to EP 5.2.12, USP, and to a lesser extent as compared with the manufacturing of a drug substance. Despite this, there are regulatory guidelines that recommend the use of therapeutic-grade raw materials whenever possible, and if not, the best available option should be used, preferably manufactured under GMP conditions. However, there is no specific GMP guidance for starting and raw materials. The recommendation is to avoid using raw materials manufactured for research use only and materials of biological origin whenever possible.

For the production of CAR-T cell products, raw materials include reagents for cell isolation, culture media, activators, and cytokines. Each

component must be carefully selected and tested to meet stringent quality standards. As research and technologies continue to advance, optimizing the preparation of raw materials for CAR-T cell products will undoubtedly contribute to improving patient outcomes and revolutionize the landscape of cancer therapy.

Cell separation reagents

The presence of certain types of cellular subgroups during the initiation of the production of CAR-T cell products can have an adverse effect on the activation and growth of T cells. The presence of myeloid derived suppressor cells and monocytes can hinder the *ex vivo* activation and expansion process of T cells. Other impurities, such as granulocytes and red blood cells, can suppress the proliferation of T cells.[11]

Hence, it is preferable that the initial material undergoes purification to ensure robust manufacturing processes. This purification can be achieved through different methods.

Using elutriation on the leukapheresis product can enhance the concentration of lymphocytes while reducing the unwanted cells by counter-flow elutriation, whereby cells align themselves based on size due to the equilibrium between centrifugal force and the flow of a buffer used to suspend the cell mixture.

Another approach involves isolating T cells or specific T cell subsets using antibody-bound magnetic beads for either positive or negative selection. This method has the potential to enhance the purity and yield of the final product and potentially improve clinical responses. This approach is possible using immunomagnetic separation on devices like the Miltenyi CliniMACS System on the CliniMACS Prodigy. This process benefits from the availability of GMP-grade monoclonal antibodies targeting the desired antigens and all the necessary raw material as buffers.[5] However, it is not only important that the reagents used for cell enrichment comply with GMP standards, but also the enrichment step, such that the reagents do not produce undesirable effects, for example, unintended cell activation that could lead to cell exhaustion. Therefore, as an alternative to the previous approach for selection, a novel, label-free T cell enrichment, a process that isolates T cells based on their biophysical properties, is being explored.[11]

Activators

In the CAR-T cell production process, activation is a fundamental step for proper genetic modification and expansion of CAR-T cells.

Co-stimulation through CD3 and a secondary signaling receptor, such as CD28, provides the wake-up signal to activate naive cells. CD3 signaling is indispensable for T cell growth, while agonistic ligation of CD28 contributes to T cell survival and plays a role in cytoskeletal remodeling, production of cytokines, differentiation, transcription, and post-translational changes during expansion.

The activation of T cells is essential for the effectiveness of the cell manufacturing process because it directly influences the efficiency of CAR transgene integration and the expansion of T cells during *ex vivo* culture.[49] In addition, the relationship between the cells, activation reagent, and the duration of the activation can have an impact over the rate of expansion, the phenotype exhibited, and, consequently, final product quality. There are some options for T cell activation, but the regulatory approval status of these reagents varies. Hence, potential users should verify their suitability for clinical manufacturing with the respective companies.

Methods for T cell activation have been developed. One of the most used is soluble anti-CD3 antibodies or immobilized monoclonal antibodies against CD3, in combination with CD28. Soluble anti-CD3 monoclonal antibodies can be used for T cell activation, but they were found to be less effective than using immobilized antibodies, and seemed to act preferentially on CD8-positive cells.

An alternative approach is coating paramagnetic beads like Dynabeads (Dynal Dynabeads: Human TActivator) with these antibodies. In suspension, the coated beads provide appropriate stimulation for considerably larger T cell cultures. In addition, this reagent enables the selection and activation in a single step when used in conjunction with the Dynal ClinExVivo MPC magnet. As the first-generation off-the-shelf clinical-grade reagent for $CD3^+$ T cell selection and activation, Dynabeads are widely used by different laboratories in early clinical trials, but before formulating the final cellular products, the beads must be eliminated to avert potential hazards during patient infusion. Bead removal is achieved by disrupting the T cell/bead aggregates through agitation, followed by passage through a robust magnetic field, which retains the beads while permitting cells to flow through.[11]

Another approach is to use other stimulation reagents, such as TransAct (Miltenyi Biotec), which uses humanized anti-CD3 and anti-CD28 antibodies conjugated to a colloidal polymeric nanomatrix. These Miltenyi particles are notably smaller, biocompatible, and are generally removed by washing before final formulation of the CART-T cell product.

These two reagents have been compared in a CD19 CAR-T cell production whereby cell expansion, phenotype, and transduction efficiency were evaluated. Cells activated with Dynabeads showed a significantly higher expansion rate as compared with TransAct, but transduction efficiency was greater for cells activated with TransAct as compared with cells activated with Dynabeads. Ultimately, a comparable yield of CD19-CAR+T cells between the two activation conditions was obtained.[50]

Successful activation has also been reported with Expamer (trademarked by Juno Therapeutics), a soluble StrepTactin protein oligomer functionalized with activating primary ligands (anti-CD3 and anti-CD28 Fab fragments) for polyclonal T cell stimulation. This reagent is soluble and can be eliminated via cell washing and provides consistent product purity.[51]

Other commercially available activation reagents include: ImmunoCult Human CD3/CD28 T cell Activator (StemCell Technologies). This

reagent comprises soluble tetrameric antibody complexes that engage CD3 and CD28 surface ligands. Moreover, alternative methodologies like soluble activation proteins, lipid microbubbles, degradable microspheres, and conjugated antibodies are under investigation as potential options.[11] Nevertheless, the manufacturer must demonstrate that possible impurities of the activator product in the final drug product have no negative impact on patient safety.

Culture medium and supplements

The selection of the most appropriate culture medium is pivotal for the production process of CAR-T cell products, as it can impact critical parameters of the final product, such as degree of cellular expansion, viability, CD4/CD8 ratio, phenotype of generated cells, or functional capacity of cells (pattern of cytokines produced and/or cytotoxic capacity). Therefore, the medium choice undoubtedly holds relevance as it influences the characteristics of the final product.

When selecting a culture medium for the manufacturing of CAR-T cell products, several considerations must be taken into account to ensure the quality of the final product (see culture medium). The media must be manufactured in accordance with applicable GMP guidelines. Moreover, it is essential to evaluate whether serum is required for manufacturing or if serum-free medium can be used. In addition to serum, other important additives in the culture media, such as pH indicators, antibiotics, and glutamine, vary between manufacturers and must be taken into account when choosing the culture medium. The best base medium should be determined by the manufacturer with demonstration as to the need for each individual component for optimal cell growth.

Other critical components required for manufacturing of CAR-T cell products are the cytokines used to promote T cell proliferation, persistence, and anti-tumor activity (see cytokines). Precise control of activation and cytokine signals are necessary to avoid potential overstimulation and cytokine release syndrome, a potentially serious complication associated with CAR-T cell therapy. In this sense, the selection of the cytokines used is of great importance and, as far as possible, only pharmaceutical grade proteins should be used.

Finally, having stability data for the medium and supplements, and functional tests that guarantee consistency between different batches, are also essential requirements.

Culture medium

Culture media serves as an essential platform for fostering the growth of T cells. These media are carefully formulated to provide the necessary support and nutrients required for optimal growth and development. Cell culture media include various components that not only serve as energy sources but also regulate crucial cellular processes. Furthermore, these media play a vital role in maintaining pH and osmolality, ensuring an ideal environment for cellular growth. A typical culture medium comprises a comprehensive array of amino acids, vitamins, inorganic salts, glucose, and (if required) proteins and hormones (e.g., insulin).

It is still common practice to grow T cells in media supplemented with serum, especially in the early phases of research and development. However, this practice can give rise to issues and complicate the regulatory approval procedure. Therefore, different companies have developed a wide range of serum-free media for culturing T cells.

A wide array of media is available on the market (see Table S2 for some examples), suitable for CAR-T cells expansion, and studies comparing different cultivation media have been published.[52]

Serum

Serum acts as a source of growth factors, hormones, and attachment factors. It provides amino acids, proteins, vitamins (particularly fat-soluble vitamins such as A, D, E, and K), carbohydrates, lipids, minerals, and trace elements. Serum can be derived from a human source or from an animal (e.g., bovine) source.

The use of serum in cell culture media has been widely adopted in scientific research and biotechnological industry due to its ability to promote cellular growth and viability. There are important considerations regarding the advantages and disadvantages associated with its use. In this text, we will explore both the benefits and potential drawbacks of using serum in culture media; alternatives to the use of serum are discussed.

Advantages of serum use in culture media:

(1) Abundance of growth factors and nutrients: Serum contains a wide array of growth factors, hormones, essential nutrients, and other bioactive components that are necessary for cell growth and survival. These elements provide a rich and balanced source of nutrients that facilitate cellular proliferation and the development of specific cellular functions.
(2) Presence of inhibitors protecting cells from a variety of conditions, such as proteolysis.
(3) Stabilizing and protective effects: Serum can possess stabilizing and protective properties that help cultured cells withstand adverse conditions, such as changes in pH, temperature, or medium composition. Moreover, it increases the viscosity of the medium and thus, protects cells from mechanical damages during agitation of suspension culture. These effects can contribute to maintaining cellular integrity and viability during long-term experiments.

Disadvantages of serum use in culture media:

(1) Variability and unknown composition: Serum is a complex biological fluid that plays a crucial role in supporting cell growth and function in culture. While the major constituents of these fluids have been identified to remain relatively constant, there are numerous minor components whose precise composition can vary significantly. However, these minor components, consisting of various nutrients, growth factors, hormones, minerals, and more, exert a significant influence on cell growth and overall

cellular behavior. Therefore, the effect of serum in cell culture can vary significantly between batches and suppliers. This variability can lead to inconsistent experimental results and difficulties in replicating studies.

(2) Risk of contamination: Serum can be a potential source of microbial, viral, or prion contamination in culture media. This can compromise experimental integrity and negatively impact results. Furthermore, the safety of the resulting CAR-T cell product may be affected.
(3) Quality assessment is imperative to ensure the suitability of each batch before its use.
(4) Increased costs of purchasing sera and for process development and maintenance.
(5) Regulatory considerations: Use of human serum is highly regulated. Compliance according to the guidelines is important for clinical trial approval.

If serum is used, the following considerations must be taken into account:

(1) Suppliers must provide the serum documentation, including a certificate of analysis.
(2) Mitigate the risk by purchasing products from approved suppliers.
(3) Decrease the risk of experimental variability attributed to interlot differences in serum by purchasing large numbers of units from a single serum batch and storing these appropriately for further use.
(4) Select the serum and the supplier to get the serum with the lowest contamination risk possible. The viral safety of materials derived from serum is detailed in section "focus on the viral safety of raw materials."

In addition, it should be noted that it is crucial to ensure the proper handling and use of serum to maximize its effectiveness and minimize potential adverse effects:

(1) Storage Conditions: Serum should be stored under proper conditions to maintain its integrity and prevent degradation. It is typically recommended to store serum at −20°C or lower temperatures to preserve its bioactivity. Avoiding repeated freeze-thaw cycles is crucial, as it can lead to protein denaturation and loss of growth-promoting factors.
(2) Serum Concentration Optimization: The optimal serum concentration in cell culture media may vary depending on the cell type, specific experimental requirements, and downstream applications. Gradual titration experiments can help determine the appropriate serum concentration that supports optimal cell growth and desired phenotypic characteristics.
(3) Viral inactivation steps in the serum process: The serum manufacturer has to implement viral inactivation steps in the serum process to inactivate potential viral contaminants. For example, a heat inactivation treatment step can be performed on serum to inactivate viral contaminants and complement before its use. Heating in an aqueous solution at 60°C for 10 h in the final container is the pharmacopeial method for virus inactivation of albumin preparations. However, heat inactivation could denature some of its constituents and adversely impact the performance of the serum. Another inactivation treatment applicable to serum is gamma irradiation. Since gamma irradiation is much less damaging to serum than heat inactivation, gamma irradiated serum can be used instead of heat-treated serum in CAR-T cell products manufacturing.
(4) Performance (suitability) testing: testing different serum batches enables researchers to identify batches that provide optimal support for their specific application, ensuring reliable and consistent results.

Historically, animal sera have been used and the most common animal serum used to supplement cell culture media has been of bovine origin. However, since the use of animal serum may be immunogenetic, human-derived supplements are preferable for clinical application. As both animal- and human-derived sera also have disadvantages, it is necessary to look for other alternatives (serum-free alternatives). The relevant guidelines for serum use are listed in Table S1.

Human serum or human plasma. Human-derived supplements provide a more biologically relevant environment for human cell culture, containing factors that are more specific to human physiology. It better mimics *in vivo* conditions, leading to more accurate cellular responses and improved translatability of experimental results.

In addition to the disadvantages of using serum mentioned previously, human serum is a limited resource and might not be available in large quantities and thus is unfavorable for commercial scale manufacturing. Furthermore, it must be taken into account that within the T cell repertoire, there are autoreactive T cells. This is important because human serum is composed almost exclusively of self-proteins, self-peptides, and self-antigens. By using human serum to culture T cells, the T cells are constantly exposed to autoantigens, which increases the possibility of activation and growth of autoreactive T cells, which can produce adverse effects. It is, therefore, necessary to look for alternatives to human serum and it is of great interest, understand serum components, and use only those factors that are beneficial.

The viral safety of materials derived from human serum or plasma is detailed in section "focus on the viral safety of raw materials."

Serum substitutes. In addition to human serum, other serum substitutes derived from human blood have been tested in the manufacturing of CAR-T cell products,[53] for example Physiologix XF, a concentrated extract from human transfusion grade whole blood fractions as a serum replacement or human platelet lysate (HPL). HPL is a common supplement used for the culture of mesenchymal stromal cells. HPL is available from some blood banks and commercial suppliers who manufacture the GMP-grade reagent (for example, STEMCELL, LSP, PL BIOSCIENCE). Recent studies

have shown that HPL can be an alternative to the use of serum for the generation of CAR-T cells, since it not only favors the genetic modification and expansion of T cells, but also results in memory cell phenotype that could promote the *in vivo* persistence of CAR-T cells.[54]

A serum-free alternative. Although the use of serum is a common practice, the advantages of cultivating clinically relevant cells without serum is a challenge that we must face; in fact, it is already recommended to avoid the use of animal sera and reduce human serum as much as possible. Consequently, the current trend is shifting toward serum-free formulations that exclude xenogeneic or human additives. These media incorporate defined quantities of purified growth factors, lipoproteins, and other proteins which are otherwise usually provided by serum. Currently, different companies have developed a wide range of serum-free media for T cells. These media are also referred to as defined culture media, since the components in these media are known. However, not all of these media comply with the relevant GMP guidelines or provide documented evidence of their purity, consistency, and stability. Therefore, before use, manufacturers of CAR-T cell products must ensure that media suppliers guarantee the quality of this raw material.

The main advantages of using serum-free media are as follows:

(1) Increased defined composition and more reproducible formulations, allowing for more convincing and consistent research results.
(2) Reduced variability and batch-to-batch dependence: The absence of serum eliminates the inherent variability and batch-to-batch dependence associated with traditional serum-containing media.
(3) Easier purification and downstream processing.
(4) Lower risk of contamination with bacteria, fungi, prions, or viruses.
(5) Easier regulatory compliance.

However, this approach also has its limitations and is therefore often unfeasible. Most serum-free formulations are specific to one cell type, so the design and formulation of serum-free media can be more complex and time consuming as compared with traditional serum-containing media. If it is decided to transition to a serum-free medium, adjustments to the protocol regarding cell seeding density, splitting routine, and activation method may be necessary, because serum-free media must be more tailored to each cell type than basic media supplemented with serum. Consequently, cell performance or final product composition could be even more dependent on the starting material. Implementing these process refinements to achieve optimal growth and maintain cell viability may require careful optimization of multiple factors, such as nutrient concentrations, growth factor combinations, and supplements. This requires a significant amount of time, generates significant additional expenses, and can cause delays. Additionally, serum-free media typically have a higher cost as compared with serum-containing media. Specialized formulations, quality control processes, and the use of recombinant growth factors contribute to increased expenditures.

Regarding the production of CAR-T cell products for clinical use, the majority of protocols still rely on serum-containing media. Transitioning away from serum-containing media to media containing these alternatives requires thorough validation and comparability studies to ensure the consistency and comparability of CAR-T cell manufacturing processes and final products. A rigorous characterization and evaluation of key parameters, such as cell phenotype, functionality, and therapeutic efficacy, are essential to establish the equivalence of CAR-T cell products based on serum alternatives or serum-free media.

Case study of comparison of serum-free culture media in CAR-T cell manufacturing

Three serum-free media (Prime-XV T cell CDM, Fujifilm, LymphoONE T cell Expansion Xeno-Free medium, Takara and TCM GMP-Prototype, CellGenix) were compared with the standard CAR-T cell medium containing fetal bovine serum (FBS). After 12 culture days, the expansion, viability, transduction efficiency, and phenotype were assessed by flow cytometry. The functionality was evaluated by intracellular staining, a chromium release assay and a long-term co-culture assay.

Expansion and viability did not differ between the CAR-T cell products generated in the three serum-free culture media compared with the standard FBS-containing medium. Intracellular cytokine staining showed that CAR-T cell products with TCM GMP-Prototype media had a statistically significant higher frequency of interferon (IFN)-γ and INF-γ^+ tumor necrosis factor-α^+ CAR-T cells than CAR-T cell products cultured with FBS. Regarding cytotoxicity evaluated by chromium release assay, CAR-T cell products generated with serum-free media showed a higher cytotoxicity than the CAR-T cell products cultured with FBS. Phenotyping assays did not reveal a significant difference in the expression of the exhaustion markers, programmed cell death protein 1, lymphocyte-activation gene 3, T cell immunoglobulin, and mucin-domain containing-3. The CAR-T cells cultured in Prime-XV T Cells CDM showed a higher percentage of central memory CAR-T cells than the CAR-T cells cultured with FBS, whereas the CAR-T cells in Prime-XV T Cells CDM and TCM GMP-Prototype had a lower frequency of naive CAR-T cells.

The present work showed that, in general, the functionality and expansion of CAR-T cells are maintained in serum-free media. Given the advantages of freedom from bovine-derived material and consistent quality, serum-free media hold promises for the future development in the field of GMP manufacturing of CAR-T cell products.[52]

Case study: CAR-T cell manufacturing using serum-free expansion media: Experiences from the Hospital Clinic and Hospital Sant Pau of Barcelona

The decision to use serum-free media for CAR-T cell products may depend on the manufacturing process selected. Thus, semi-automated cell manufacturing like CliniMACS Prodigy may allow to generate CAR-T cell products successfully without the use of any serum source. Comparative studies with human T cells have demonstrated that this platform is able to consistently generate CAR-T cell products using

Table 5. Advantages and disadvantages of using IL-2 versus IL-7+IL-15 in CAR-T cell expansion

IL-2	Disadvantages	Low enhanced migration ability.
		Less anti-tumor activity.
		Cells are expanded slower and have more apoptotic and senescence markers and activity.
	Advantages	Enhance the CD8$^+$ effector memory T cell population *in vitro*.
IL-7+ IL-15	Disadvantages	No differences in CAR transduction efficiency.
		No significant difference in immune cytokine release (IL-2, IFN-γ, tumor necrosis factor-α) or specific lysis.
	Advantages	Instruct T cells toward memory stem cell-like phenotypes, which are less differentiated and have a superior capacity for expansion and survival.
		Cells more efficiently expanded.
		Higher proliferation and lower apoptosis rate (higher expression of the anti-apoptosis protein BCL-2).
		Superior anti-tumour activity *in vivo*.
		CD8$^+$ CAR-T cell expansion is enhanced.
		Increase of the CD8$^+$ naive T cell and central memory T cell populations *in vitro*.
		Higher expression levels of chemokine receptors CCR7 and CXCR4.
		Enhanced migration ability shown.

Reviewed from Zhou et al. 2019.[60]

serum-free media that were compliant with GMP conditions for clinical use. Importantly, compared with CAR-T cell products generated with medium containing human serum, those CAR-T cell products generated with serum-free media had a comparable *ex vivo* expansion and *in vitro* and *in vivo* antitumor function that CAR-T cell products manufactured with media-containing human serum. Furthermore, the final product generated under serum-free conditions had comparable cell numbers and viability, as well as T cell subset composition. In our experience at the Hospital Sant Pau in Barcelona, two academic CAR-T cell products (HSP-CAR30 and HSP-CAR19M) have been manufactured successfully with serum-free media and infused into patients with refractory hematological malignancies (Hodgkin and non-Hodgkin B cell lymphoma) in two independent clinical trials (NCT04653649 and EudraCT 2020-3133-38).[55] CAR-T cell products were manufactured successfully in all cases, allowing the infusion of up to 10 × 10e6 CAR-T cells/kg.

The Hospital Clinic of Barcelona—IDIBAPS has developed two academic products: ARI0001 (Varnimcabtagene autoleucel) and ARI0002h (Cesnicabtagene autoleucel). ARI0001 was developed with human serum supplemented TexMACS media, while ARI0002h was developed with human serum-free TexMACS media. Both products have been tested for safety and efficacy in their corresponding phase 1 clinical trials (NCT04309981 and NCT03144583) and demonstrated that both products could be manufactured with both approaches, leading to an effective and secure CAR-T cell product.[56,57]

Collectively, these data support the use of serum-free media for manufacturing CAR-T cell products, at least when using a dedicated platform such as the CliniMACS Prodigy. However, this approach should be carefully evaluated when other cell manufacturing devices are used.

Cytokines

CAR-T cell products need to be expanded *ex vivo* for clinical use. Typically, T cell expansion is the longest and one of the most critical phases in the CAR-T workflow, as engineered cells are sensitive to their micro-environment and growth conditions. Cytokines used for the expansion influence the composition, quality and phenotype of CAR-T cells. After the expansion, the number of cells for the patient treatment must be reached while maintaining the viability. Also, less differentiated memory cell phenotypes (younger cells) are interesting because they result in more functionality and effective therapeutic outcomes. The CD8:CD3 ratio in the infusion product is also dependent on supplements added to the T cell expansion medium.

There are some cytokines that are commonly used for CAR-T cell expansion: IL-2, IL-7, IL-15, and IL-21, the most commonly used combination being IL-2 or IL-7 with or without IL-15.[58]

IL-2 is one of the most well-known cytokines used in T cell expansion. It promotes T cell growth, survival, and proliferation. However, the cells expanded with IL-2 have a maturated terminal effector T cell phenotype with high functional capacity, but reduced persistence in blood. In addition, IL-2 favors the expansion of regulatory T cells that can inhibit the antitumor activity of the CAR-T cells (Table 5).[59]

The expansion with IL-7 enhances activation and proliferation when compared with IL-2, but reduces the CAR-T cells' terminal differentiation. The combination of IL-7 and IL-15 also promotes the survival and maintenance of less differentiated T cells, such as naive T cells and T stem cell memory cells and increases the percentage of memory cells in the final cellular suspension, yielding improved *in vivo* persistence[59] that correlates with CAR-T cells more effective *in vivo* (Table 5).

IL-21 has immunostimulatory effects on T cells and can promote their proliferation, differentiation, and effector functions. It may enhance the anti-tumor activity of CAR T cells. It is demonstrated that IL-21 in combination with IL-2, IL-7, or IL-15, enhances T cell transfection, enrichment, and expansion of less differentiated CAR-T cells and improves the CAR-T cell cytotoxicity, which was related to an increased secretion of effector cytokines.[61]

Despite the importance of the cytokines in the expansion having a significant impact on the behavior and characteristics of CAR T cells, the optimal cytokine composition is not yet clearly defined and researchers must optimize the conditions for their manufacturing protocols.

The cytokines used for the production of cell- or gene therapy-based products are produced by recombinant DNA technology. The production protocol must guarantee that the presence of

impurities in the final product is be reduced to acceptable levels. Identity, purity, and the biological activity must be demonstrated by specific assays such as liquid chromatography or proliferation assays. The endotoxin content and the sterility have to be analyzed in compliance with the European Pharmacopeia.

Other supplements

Glutamine. L-Glutamine is an essential amino acid that is a crucial component of culture media and serves as a major energy source for the cells, especially when rapidly dividing. Many culture media contain L-glutamine, but as glutamine is an unstable amino acid, most media are formulated without glutamine in the basal formula. Glutamine is then subsequently added before use. L-Glutamine solutions manufactured under GMP conditions are available on the market to be used in culture media.

Antibiotics. The routine use of antibiotics for cell cultures in the preparation of advanced therapy drugs is not recommended. Antibiotics can interfere with the metabolism of sensitive cells. Fundamentally, antibiotics can mask contamination by mycoplasma and bacteria and can interfere with the sterility testing. Furthermore, the risk of an allergic reaction by the recipient remains a safety concern.

Focus on the viral safety of raw materials

To limit the inherent risk of introducing viral contaminants in the manufacturing process of CAR-T cell products, the following measures have to be implemented for the selection of raw materials:

(1) By first intent, it is recommended to avoid, whenever possible, the use of materials of biological origin.
(2) If materials of biological origin are required for production, appropriate measures must be taken to minimize the risks of transmitting adventitious agents (viruses and prions). It is the responsibility of the manufacturer to ensure that the viral safety of each material is ensured and that the required viral safety documentation is available for conventional viruses and prions (TSE agent).

The viral safety requirements for materials of animal origin, materials of human origin and biotechnology products are detailed below.

The viral safety requirements for materials of animal origin (e.g., materials derived from animal serum) include the following:

(1) Information on the source: species, organ/tissue used, country of origin, animals' health status (animals fulfilling specific health requirements, fit for human consumption, and reared under controlled conditions, when applicable).
(2) Testing for the detection of viral contaminants performed on the animals and/or on the material. (The viral contaminant tests to be performed depend on the material origin.)
(3) Inactivation treatment performed on the material whenever possible (e.g., gamma irradiation, heat inactivation, pasteurization) and viral clearance studies (virus validation studies) performed for this treatment (if applicable).
(4) BSE/TSE statements, if applicable.

The viral safety requirements for materials of human origin (e.g., materials derived from human serum/plasma) include the following:

(1) Information on the source: organ/tissue used, donors' country of origin, and information on donors' eligibility/screening. For human blood- and tissue-derived materials, only carefully evaluated donors who have been adequately tested for infectious transmissible agents may be used.
(2) Testing for viral contaminants performed on donors, donations, and pools.
(3) Information on the number of donations pooled. (Due to the inherent risk of transmitting infectious agents from materials derived from pooled plasma/sera, consideration is given to limit the number of donations which are pooled, unless sufficient methods for inactivation/removal of viruses are applied during production, where applicable.)
(4) Inactivation treatment performed on the material whenever possible (e.g., irradiation, heat inactivation, pasteurization) and viral clearance studies (virus validation studies) performed for this treatment, if applicable.
(5) Reference of Plasma Master File if available (to be included in regulatory submissions).
(6) Compliance with appropriate EU and/or national legislation applicable to transplantation and transfusion.
(7) Traceability measures to enable each donation to be followed from donation to raw material and to the final product, and vice versa.

For biotechnology products prepared on a cell line of human or animal origin (such as recombinant proteins and antibodies), their viral safety must comply with the requirements of the ICH Q5A (R2) guideline and is based on a combination of the following measures:

(1) Careful selection of the cell line and materials used in the manufacturing process: All the materials of biological origin used must be identified (including materials used for cell line development, cell banking, production and formulation), and viral safety data must be available for each material of biological origin, in regard to conventional viruses and prions (TSE agent).
(2) Testing for the detection of viral contaminants performed at different stages, in particular on cell banks, process intermediates and/or on the product.
(3) Viral clearance steps included in the manufacturing process, namely, steps effective to remove or inactivate potential viral contaminants (e.g., low pH treatment, detergent treatment, nanofiltration, and chromatography steps).
(4) Viral clearance studies (virus validation studies) performed to demonstrate the effectiveness of these viral clearance steps.

Regulatory consideration for raw materials

GMP grade

One of the critical factors influencing the safety and efficacy of cell and gene therapy products is the use of high-quality GMP raw materials, manufactured to strict quality standards and rigorously tested to

Table 6. Applicable guidelines and directives for supplier qualification

Guideline/Directive/Reference	Topics
EU-GMP Guidelines Chapter 5 (Production), 5.27	"The selection, qualification, approval and maintenance of suppliers of starting materials, together with their purchase and acceptance, should be documented as part of the pharmaceutical quality system"
EU-GMP Guidelines Chapter 5 (Production), 5.29	"Audits should be carried out at the manufacturers and distributors of active substances to confirm that they comply with the relevant good manufacturing practice and good distribution practice requirements."

ensure their safety and suitability for use in clinical trials and commercial production.

Since the use of non-GMP-grade materials can lead to unexpected results that may affect the quality, safety, and efficacy of the final product, the use of GMP-grade materials reduces the likelihood of complications during the manufacturing process, such as batch-to-batch variability and impurities, and is essential to avoid additional risk assessments, comparability runs, changes in material suppliers during development, and extensive discussions with regulatory authorities.

Such bodies, like the US Food and Drug Administration (FDA), the EMA, and other national regulatory authorities, require extensive documentation of the materials used in the manufacturing process. The use of GMP-grade materials helps to streamline the regulatory approval process by reducing the need for additional testing and validation. In addition, the use of GMP-grade materials provides other benefits such as improved product quality. GMP materials are manufactured in a controlled environment, minimizing the risk of contamination, and ensuring batch-to-batch consistency.

Manufacturers of GMP-grade material should provide comprehensive documentation outlining the raw materials used, quality testing methods and results, impurities, stability, and, if required, viral safety (for the materials of biological origin). Manufacturers must prioritize the use of high-quality GMP-grade materials and adhere to strict regulatory compliance to provide safe and effective therapies to patients. Failure to do so can result in delays in the regulatory approval process, additional costs and can have a negative impact on patient safety and outcomes.

To ensure the supply of the right GMP-compliant materials, and thus the long-term production of the CAR-T cell products being developed, a careful and comprehensive selection process is required to identify suitable manufacturers and backup manufacturers.

Selection of supplier

During the development and production of ATMPs, the selection and sourcing of raw materials play a critical role. Concerning the European Union, the EU-GMP Guidelines Chapter 5 (Production), 5.27 applies to issues related to suppliers and raw material (Table 6). An integrated supplier qualification process is essential for identifying and mitigating risks associated with the supplied materials, components, and services.[62]

When starting the supplier qualification process, it is crucial to identify and specify the regulatory requirements applicable to the type of material, component or service and the specific product in question. Supplier audits, which must be carefully planned and carried out, are crucial to the success of supplier selection. Audits serve as a valuable risk assessment tool, instilling confidence that suppliers, vendors and contractors possess the ability to consistently deliver materials, components and services of the required quality, while respecting regulatory standards. However, supplier qualification goes beyond mere auditing. Achieving a successful audit does not conclude the qualification process. In addition, even after finalizing the supplier selection and establishing the contract, the selected suppliers must be periodically evaluated for compliance with the relevant requirements and must be carefully evaluated to ensure that there are no changes related to the facilities or the manufacturing process that may affect compliance with the required requirements.

It is also important to note that, in addition to the supplier, the brokers involved in the process should undergo qualification, considering the provisions described in the Guidelines on Good Distribution Practices of medicines for human use (2013/C 343/01), Chapter 10, Specific provisions for Runners.

To gain a thorough comprehension of a product and its manufacture, it is important to create a precise list of questions (also known as a supplier questionnaire) that cover a range of relevant topics that are shown in Table S3. Moreover, other topics categorized into groups, such as business, facility, quality, and expertise, should also be taken into account (Table S4). For each product, it is crucial to ask the following questions: What are the specific requirements in terms of quality, quantity, and price? Is there sufficient time available to meet these requirements? Additionally, it is important to consider the next steps in the manufacturing process, such as the ability to scale up production with the same or higher quality, either already in place or planned for the future.

Based on the questions, which are essential for defining the manufacturing strategy, each Contract Manufacturing Organization (CMO)/provider can be classified and assigned a score (indicating the "best" to "unsatisfying" response) based on their answers. This classification should be initially based on objective and pragmatic criteria. For example, did the provider/CMO respond partially, fully, or not at all?

By assessing the satisfaction level of the answers, including whether they are complete, partial, or out of scope, and incorporating the score per question, along with the weight assigned to important aspects for a product, it becomes possible to rank the CMO/providers. Furthermore, this evaluation process helps identify potential risks, unanswered questions, and strategies required for the product's manufacturing and future support.

With regard to efficiency both for developers and suppliers, the development of centralized audits with harmonized qualification documentation by DARE-NL as the Dutch platform for ATMP Research and the Dutch-Belgian ATMP working group, is exemplary (see https://www.dare-nl.nl/activities/#wp2). The qualification documentation, audit reports and card files for qualified starting and raw materials from the suppliers is filed centrally in the DARE-NL.Zenya IT system. The QA department of each affiliated DARE-NL and ATMP working group member is entitled to review and judge the audit report for qualification of the supplier. This centralized (re)-qualification procedure decreases the labor-intensive qualification procedure both for developers, as well as suppliers. Ultimately, many other cell and gene therapy networks, or even manufacturing facilities in an entire country, could benefit from such an approach.

Change in raw material during the development and regulatory requirements

In certain situations, changes in raw material may become necessary due to various factors, such as availability, cost, quality, or regulatory compliance. Any change in raw materials has the potential to affect the final product formulation, stability, bioavailability, and overall performance. Therefore, any change must be justified, and the modified final product must meet the established requirements.

Reasons for changing raw materials. The raw materials used in the CAR-T product manufacturing process may have to be changed during development or after the marketing authorization in several cases.

(1) Supply chain considerations: In some cases, a change in the raw material may be necessitated by issues in the supply chain, such as discontinuation, scarcity, or unavailability of the original raw material.
(2) Cost optimization: Raw material costs can significantly impact the overall manufacturing expenses. Switching to alternative raw materials that are cost effective while maintaining the required quality standards may be considered to enhance cost optimization strategies.
(3) Quality improvement: If a manufacturer identifies a raw material that exhibits better quality attributes, switching to this alternative material may be desirable to improve the drug product's performance, stability, or safety profile. A raw material can also be changed following a suggestion from competent authorities (e.g., if the quality or viral safety of the material initially used is not optimal).

Regulatory requirements and considerations. Change in raw materials during drug development and production is a complex process that requires careful evaluation, adherence to regulatory requirements, and a focus on maintaining product quality and safety.

Key considerations and requirements:

(1) Regulatory authority notifications: Depending on the specific regulatory guidelines, manufacturers may be required to notify the appropriate regulatory authorities about any significant changes in raw materials. In the EU, this typically involves submitting a variation if the product is already approved or an amendment if clinical trials are on-going, providing comprehensive information on the new material used, justification of the change, and its impact on the final drug product.
(2) Comparability studies: To support a significant change in raw materials, manufacturers are generally required to conduct comparability studies. These studies aim to demonstrate that the raw material change has not had an adverse impact on the quality, efficacy and/or safety profile of the final product. Analytical testing, stability studies, and bioequivalence assessments may be necessary to establish comparability following current guidelines:

   ○ ICH Q5E (https://www.ema.europa.eu/en/ich-q5e-biotechnological-biological-products-subject-changes-their-manufacturing-process).

   ○ FDA draft guideline: Manufacturing Changes and Comparability for Human Cellular and Gene Therapy Products (https://www.fda.gov/regulatory-information/search-fda-guidance-documents/manufacturing-changes-and-comparability-human-cellular-and-gene-therapy-products).

   ○ EMA Q&A: Questions and answers on comparability considerations for ATMP - Scientific guideline (https://www.ema.europa.eu/en/questions-answers-comparability-considerations-advanced-therapy-medicinal-products-atmp-scientific).
(3) Documentation and reporting: All changes in raw materials should be thoroughly documented and maintained in accordance with GMP requirements. Manufacturers must keep detailed records of the change, including the rationale, supporting data, and any associated risk assessments.
(4) Risk assessment: Manufacturers should perform a comprehensive risk assessment to evaluate the impact of raw material changes, taking into account the cumulative effect that the sum of small changes may have.

Due to the implications on the final product, manufacturers must properly evaluate the need for raw material changes. Close collaboration between research and development teams, quality control, quality assurance, and regulatory affairs departments is essential to successfully navigate the challenges associated with changing raw materials.

CONCLUSION

The GMP production of CAR-T cell products requires careful selection of starting and raw materials. The aim of the publication was to summarize important aspects for the selection of these materials. Requirements and aspects of leukapheresis selection and testing were presented. The production of the vector was described, including release specifications. The commonly used raw and starting materials were described with regard to their impact on the manufacture of

CAR-T cell products and important aspects of selecting the right material were explained. Particular attention was paid to viral safety of all raw and starting materials of biological origin. The various directives and regulations applicable to ATMPs define specific expectations that must be met. These have been summarized in the appendix to assist all new GMP manufacturers in the selection of materials and suppliers.

This publication underlines the vision of the Innovative Medicines Initiative, T^{2}EVOLVE, to help developers accelerate the development of CAR-T cell therapies in the EU, ensure the manufacturing of safe and effective products, and to increase the awareness and access to these innovative immunotherapies for cancer patients.

ACKNOWLEDGMENTS

The T^{2}EVOLVE consortium was formed in the beginning of 2021 with the aim to accelerate the development of and increase access to CAR- and TCR-engineered T cell therapies. This European Innovative Medicine Initiative (IMI) consortium is a highly interactive multi-disciplinary and multi-stakeholder, public and private partnership that brings together key opinion leaders in engineering T cell therapies, adult and pediatric hematology and oncology, translational research and regulation, patient advocacy, data management, biotech and pharmaceutical industries, health economics, and health technology assessment (HTA). The authors would also like to thank Trudy Straetemans and Coba van Zanten of the Dutch infrastructure for cancer-specific ATMP research (DARE-NL), a unique partnership between all academic developers of advanced therapy medicinal products (ATMPs) in the Netherlands, for the great discussion on a centralized way to qualify raw material suppliers and for their support of this publication. Furthermore, the authors confirm that financial support was received for the research, authorship, and/or publication of this article. This project has received funding from the Innovative Medicines Initiative 2 Joint Undertaking under grant agreement No 945393. This Joint Undertaking receives support from the European Unionś Horizon 2020 research and innovation program and EFPIA and EUROPEAN HEMATOLOGY ASSOCIATION.

AUTHOR CONTRIBUTIONS

All authors wrote the original draft and were involved in review and editing. M.L. took care of the visualization.

DECLARATION OF INTERESTS

C.M., S.A., and H.N. were employed by the Institut de Recherches Internationales Servier. T.T. and D.A. were employed by Astellas. R.C. was employed by Takeda Pharmaceuticals. I.S. was employed by Bayer Vital GmbH. B.S. was employed by Miltenyi Biotec B.V. & Co. KG.

SUPPLEMENTAL INFORMATION

Supplemental information can be found online at https://doi.org/10.1016/j.ymthe.2024.11.017.

REFERENCES

1. Directive 2001/83/EC of the European Parliament and of the Council of 6 November 2001 on the Community code relating to medicinal products for human use. (p. 133–188). https://eur-lex.europa.eu/eli/dir/2001/83/2022-01-01.
2. European Directorate for the Quality of Medicines & HealthCare. European Pharmacopoeia. Ph. Eur. 5.2.12: raw materials of biological origin for the production of cell based and gene therapy medicinal products. https://www.edqm.eu/en/european-pharmacopoeia-ph.-eur.-11th-edition.
3. Belcastro, L. (2020). Raw material qualification and optimization: the Janssen approach. Cell Gene Ther. Insights *6*, 235–240.
4. Belcastro, L., Brabec, M., Clarke, L., Luke, R., and Tomtishen III, J.P. (2021). Fitting product to process: raw materials customization for cell therapy manufacturing success. Cell Gene Ther. Insights *7*, 183–198.
5. Gee, A.P. (2018). GMP CAR-T cell production. Best practice & research. Clin. Haematol. *31*, 126–134.
6. IPRP. International Pharmaceutical Regulators Programme. https://www.iprp.global/home.
7. European Medicines Agency. ICH Q9 Quality risk management - Scientific guideline. https://www.ema.europa.eu/en/ich-q9-quality-risk-management-scientific-guideline.
8. Gauthy, E., Lambricht, L., and Snykers, S. (2019). How to effectively manage critical raw materials to support clinical development in CAR-T cell therapy. Cell Gene Ther. Insights *5*, 133–140.
9. Carabello, J. (2020). The role of raw materials in the manufacturing of cell and gene therapy medicinal products: practices and challenges. Cell Gene Ther. Insights *2020*, 229–233.
10. RegMedNet and CellGenix. Raw Materials for CAR-T Production. https://www.regmednet.com/ebook-raw-materials-for-car-t-production.
11. Abou-El-Enein, M., Elsallab, M., Feldman, S.A., Fesnak, A.D., Heslop, H.E., Marks, P., Till, B.G., Bauer, G., and Savoldo, B. (2021). Scalable Manufacturing of CAR T cells for Cancer Immunotherapy. Blood Cancer Discov. *2*, 408–422.
12. Li, Y., Huo, Y., Yu, L., and Wang, J. (2019). Quality Control and Nonclinical Research on CAR-T Cell Products: General Principles and Key Issues. Engineering *5*, 122–131.
13. Nezvalova-Henriksen, K., Langebrake, C., Bauters, T., Moreno-Martinez, M.-E., Ahnfelt, E., Ekelund, H., Domingos, V., Pires, V., Bonnin, A., Bojanić, I., et al. (2023). Implementation and operational management of marketed chimeric antigen receptor T cell (CAR-T Cell) therapy-a guidance by the GoCART Coalition Pharmacist Working Group. Bone Marrow Transpl. *58*, 1069–1074.
14. Bartoló-Ibars, A., Uribe-Herranz, M., Muñoz-Sánchez, G., Arnaldos-Pérez, C., Ortiz-Maldonado, V., Urbano-Ispizua, Á., Pascal, M., and Juan, M. (2021). CAR-T after Stem Cell Transplantation in B-Cell Lymphoproliferative Disorders: Are They Really Autologous or Allogenic Cell Therapies? Cancers *13*, 4664.
15. Ruella, M., Xu, J., Barrett, D.M., Fraietta, J.A., Reich, T.J., Ambrose, D.E., Klichinsky, M., Shestova, O., Patel, P.R., Kulikovskaya, I., et al. (2018). Induction of resistance to chimeric antigen receptor T cell therapy by transduction of a single leukemic B cell. Nat. Med. *24*, 1499–1503.
16. Allen, E.S., Stroncek, D.F., Ren, J., Eder, A.F., West, K.A., Fry, T.J., Lee, D.W., Mackall, C.L., and Conry-Cantilena, C. (2017). Autologous lymphapheresis for the production of chimeric antigen receptor T cells. Transfusion *57*, 1133–1141.
17. Caldwell, K.J., Gottschalk, S., and Talleur, A.C. (2020). Allogeneic CAR Cell Therapy-More Than a Pipe Dream. Front. Immunol. *11*, 618427.
18. Tyagarajan, S., Schmitt, D., Acker, C., and Rutjens, E. (2019). Autologous cryopreserved leukapheresis cellular material for chimeric antigen receptor-T cell manufacture. Cytotherapy *21*, 1198–1205.
19. Meneghel, J., Kilbride, P., and Morris, G.J. (2020). Cryopreservation as a Key Element in the Successful Delivery of Cell-Based Therapies-A Review. Front. Med. *7*, 592242.
20. Elavia, N. (2017). The Post-Thaw Recovery of Cryopreserved Chimeric Antigen Receptor (CAR) T-Cells during Manufacture Is Better Than That of Cryopreserved Peripheral Blood CD3+ Cells. Blood *130*, 4475.
21. Wang, X., and Rivière, I. (2016). Clinical manufacturing of CAR T cells: foundation of a promising therapy. Mol. Ther. Oncolytics *3*, 16015.
22. Xu, L., Chen, H., Chen, J., Han, M., Huang, H., Lai, Y., Liu, D., Liu, Q., Liu, T., Jiang, M., et al. (2018). The consensus on indications, conditioning regimen, and donor selection of allogeneic hematopoietic cell transplantation for hematological diseases in China-recommendations from the Chinese Society of Hematology. J. Hematol. Oncol. *11*, 33.
23. Castella, M., Boronat, A., Martín-Ibáñez, R., Rodríguez, V., Suñé, G., Caballero, M., Marzal, B., Pérez-Amill, L., Martín-Antonio, B., Castaño, J., et al. (2019). Development of a Novel Anti-CD19 Chimeric Antigen Receptor: A Paradigm for an Affordable CAR T Cell Production at Academic Institutions. Mol. Ther. Methods Clin. Dev. *12*, 134–144.
24. Monjezi, R., Miskey, C., Gogishvili, T., Schleef, M., Schmeer, M., Einsele, H., Ivics, Z., and Hudecek, M. (2017). Enhanced CAR T-cell engineering using non-viral Sleeping Beauty transposition from minicircle vectors. Leukemia *31*, 186–194.
25. Hudecek, M., and Ivics, Z. (2018). Non-viral therapeutic cell engineering with the Sleeping Beauty transposon system. Curr. Opin. Genet. Dev. *52*, 100–108.

26. Ivics, Z., and Izsvák, Z. (2010). The expanding universe of transposon technologies for gene and cell engineering. Mob. DNA *1*, 25.

27. Kebriaei, P., Singh, H., Huls, M.H., Figliola, M.J., Bassett, R., Olivares, S., Jena, B., Dawson, M.J., Kumaresan, P.R., Su, S., et al. (2016). Phase I trials using Sleeping Beauty to generate CD19-specific CAR T cells. J. Clin. Invest. *126*, 3363–3376.

28. Harmening, N., Johnen, S., Izsvák, Z., Ivics, Z., Kropp, M., Bascuas, T., Walter, P., Kreis, A., Pajic, B., and Thumann, G. (2023). Enhanced Biosafety of the Sleeping Beauty Transposon System by Using mRNA as Source of Transposase to Efficiently and Stably Transfect Retinal Pigment Epithelial Cells. Biomolecules *13*, 658.

29. Prommersberger, S., Monjezi, R., Botezatu, L., Miskey, C., Amberger, M., Mestermann, K., Hudecek, M., and Ivics, Z. (2022). Generation of CAR-T Cells with Sleeping Beauty Transposon Gene Transfer. Methods Mol. Biol. *2521*, 41–66.

30. Magnani, C.F., Myburgh, R., Brunn, S., Chambovey, M., Ponzo, M., Volta, L., Manfredi, F., Pellegrino, C., Pascolo, S., Miskey, C., et al. (2023). Anti-CD117 CAR T cells incorporating a safety switch eradicate human acute myeloid leukemia and hematopoietic stem cells. Mol. Ther. Oncolytics *30*, 56–71.

31. Prommersberger, S., Monjezi, R., Shankar, R., Schmeer, M., Hudecek, M., Ivics, Z., and Schleef, M. (2022). Minicircles for CAR T Cell Production by Sleeping Beauty Transposition: A Technological Overview. Methods Mol. Biol. *2521*, 25–39.

32. Prommersberger, S., Reiser, M., Beckmann, J., Danhof, S., Amberger, M., Quade-Lyssy, P., Einsele, H., Hudecek, M., Bonig, H., and Ivics, Z. (2021). CARAMBA: a first-in-human clinical trial with SLAMF7 CAR-T cells prepared by virus-free Sleeping Beauty gene transfer to treat multiple myeloma. Gene Ther. *28*, 560–571.

33. Cary, L.C., Goebel, M., Corsaro, B.G., Wang, H.G., Rosen, E., and Fraser, M.J. (1989). Transposon mutagenesis of baculoviruses: analysis of Trichoplusia ni transposon IFP2 insertions within the FP-locus of nuclear polyhedrosis viruses. Virology *172*, 156–169.

34. Wilson, M.H., Coates, C.J., and George, A.L. (2007). PiggyBac transposon-mediated gene transfer in human cells. Mol. Ther. *15*, 139–145.

35. Li, M.A., Turner, D.J., Ning, Z., Yusa, K., Liang, Q., Eckert, S., Rad, L., Fitzgerald, T.W., Craig, N.L., and Bradley, A. (2011). Mobilization of giant piggyBac transposons in the mouse genome. Nucleic Acids Res. *39*, e148.

36. Yusa, K., Zhou, L., Li, M.A., Bradley, A., and Craig, N.L. (2011). A hyperactive piggyBac transposase for mammalian applications. Proc. Natl. Acad. Sci. USA *108*, 1531–1536.

37. Nakazawa, Y., Huye, L.E., Dotti, G., Foster, A.E., Vera, J.F., Manuri, P.R., June, C.H., Rooney, C.M., and Wilson, M.H. (2009). Optimization of the PiggyBac transposon system for the sustained genetic modification of human T lymphocytes. J. Immunother. *32*, 826–836.

38. Bishop, D.C., Xu, N., Tse, B., O'Brien, T.A., Gottlieb, D.J., Dolnikov, A., and Micklethwaite, K.P. (2018). PiggyBac-Engineered T Cells Expressing CD19-Specific CARs that Lack IgG1 Fc Spacers Have Potent Activity against B-ALL Xenografts. Mol. Ther. *26*, 1883–1895.

39. Nakazawa, Y., Matsuda, K., Kurata, T., Sueki, A., Tanaka, M., Sakashita, K., Imai, C., Wilson, M.H., and Koike, K. (2016). Anti-proliferative effects of T cells expressing a ligand-based chimeric antigen receptor against CD116 on CD34(+) cells of juvenile myelomonocytic leukemia. J. Hematol. Oncol. *9*, 27.

40. Nakazawa, Y. (2022). A first-in-human clinical trial of piggyBac transposon-mediated GMR CAR-T cells against CD116-positive acute myeloid leukemia and juvenile myelomonocytic leukemia. Rinsho. Ketsueki. *63*, 776–781.

41. Yagyu, S., and Nakazawa, Y. (2023). piggyBac-transposon-mediated CAR-T cells for the treatment of hematological and solid malignancies. Int. J. Clin. Oncol. *28*, 736–747.

42. Zhang, Y., Zhang, Z., Ding, Y., Fang, Y., Wang, P., Chu, W., Jin, Z., Yang, X., Wang, J., Lou, J., and Qian, Q. (2021). Phase I clinical trial of EGFR-specific CAR-T cells generated by the piggyBac transposon system in advanced relapsed/refractory non-small cell lung cancer patients. J. Cancer Res. Clin. Oncol. *147*, 3725–3734.

43. Miliotou, A.N., and Papadopoulou, L.C. (2020). Correction to: In Vitro-Transcribed (IVT)-mRNA CAR Therapy Development. Methods Mol. Biol. *2086*, C1.

44. Kowalski, P.S., Rudra, A., Miao, L., and Anderson, D.G. (2019). Delivering the Messenger: Advances in Technologies for Therapeutic mRNA Delivery. Mol. Ther. *27*, 710–728.

45. Beatty, G.L., O'Hara, M.H., Lacey, S.F., Torigian, D.A., Nazimuddin, F., Chen, F., Kulikovskaya, I.M., Soulen, M.C., McGarvey, M., Nelson, A.M., et al. (2018). Activity of Mesothelin-Specific Chimeric Antigen Receptor T Cells Against Pancreatic Carcinoma Metastases in a Phase 1 Trial. Gastroenterology *155*, 29–32.

46. Krug, C., Wiesinger, M., Abken, H., Schuler-Thurner, B., Schuler, G., Dörrie, J., and Schaft, N. (2014). A GMP-compliant protocol to expand and transfect cancer patient T cells with mRNA encoding a tumor-specific chimeric antigen receptor. Cancer Immunol. Immunother. *63*, 999–1008.

47. Wiesinger, M., März, J., Kummer, M., Schuler, G., Dörrie, J., Schuler-Thurner, B., and Schaft, N. (2019). Clinical-Scale Production of CAR-T Cells for the Treatment of Melanoma Patients by mRNA Transfection of a CSPG4-Specific CAR under Full GMP Compliance. Cancers *11*, 1198.

48. Kitte, R., Rabel, M., Geczy, R., Park, S., Fricke, S., Koehl, U., and Tretbar, U.S. (2023). Lipid nanoparticles outperform electroporation in mRNA-based CAR T cell engineering. Molecular therapy. Mol. Ther. Methods Clin. Dev. *31*, 101139.

49. Ayala Ceja, M., Khericha, M., Harris, C.M., Puig-Saus, C., and Chen, Y.Y. (2024). CAR-T cell manufacturing: Major process parameters and next-generation strategies. J. Exp. Med. *221*, e20230903.

50. Mock, U., Nickolay, L., Philip, B., Cheung, G.W.-K., Zhan, H., Johnston, I.C.D., Kaiser, A.D., Peggs, K., Pule, M., Thrasher, A.J., and Qasim, W. (2016). Automated manufacturing of chimeric antigen receptor T cells for adoptive immunotherapy using CliniMACS prodigy. Cytotherapy *18*, 1002–1011.

51. Poltorak, M.P., Graef, P., Tschulik, C., Wagner, M., Cletiu, V., Dreher, S., Borjan, B., Fraessle, S.P., Effenberger, M., Turk, M., et al. (2020). Expamers: a new technology to control T cell activation. Sci Rep *10*, 17832.

52. Eberhardt, F., Hückelhoven-Krauss, A., Kunz, A., Jiang, G., Sauer, T., Reichman, A., Neuber, B., Böpple, K., Schmitt, A., Müller-Tidow, C., et al. (2023). Impact of serum-free media on the expansion and functionality of CD19.CAR T-cells. Int. J. Mol. Med. *52*, 58.

53. Watanabe, N., Mo, F., and McKenna, M.K. (2022). Impact of Manufacturing Procedures on CAR T Cell Functionality. Front. Immunol. *13*, 876339.

54. Canestrari, E., Steidinger, H.R., McSwain, B., Charlebois, S.J., and Dann, C.T. (2019). Human Platelet Lysate Media Supplement Supports Lentiviral Transduction and Expansion of Human T Lymphocytes While Maintaining Memory Phenotype. J. Immunol. Res. *2019*, 3616120.

55. Caballero Gonzalez, A.C.C., Escribà-García, L., Montserrat, R., Escudero-López, E., Pujol-Fernández, P., Ujaldón-Miró, C., García-Cadenas, I., Esquirol, A., Martino, R., Sierra, J., et al. (2022). Phase 1 Clinical Trial of Memory-Enriched Academic HSP-CAR30 for the Treatment of Relapsed/Refractory Hodgkin Lymphoma and CD30+ T-Cell Lymphoma: Clinical and Biological Studies. Blood *140*, 405–407.

56. Castella, M., Caballero-Baños, M., Ortiz-Maldonado, V., González-Navarro, E.A., Suñé, G., Antoñana-Vidósola, A., Boronat, A., Marzal, B., Millán, L., Martín-Antonio, B., et al. (2020). Point-of-care CAR T-cell production (ARI-0001) using a closed semi-automatic bioreactor: experience from an academic phase I clinical trial. Front immuno *11*, 00482.

57. Oliver-Caldés, A., González-Calle, V., Cabañas, V., Español-Rego, M., Rodríguez-Otero, P., Reguera, J.L., López-Corral, L., Martin-Antonio, B., Zabaleta, A., Inogés, S., et al. (2023). Fractionated initial infusion and booster dose of ARI0002h, a humanised, BCMA-directed CAR T-cell therapy, for patients with relapsed or refractory multiple myeloma (CARTBCMA-HCB-01): a single-arm, multicentre, academic pilot study. Lancet Oncol *24*, 913–924.

58. Stock, S., Schmitt, M., and Sellner, L. (2019). Optimizing Manufacturing Protocols of Chimeric Antigen Receptor T Cells for Improved Anticancer Immunotherapy. Int. J. Mol. Sci. *20*, 6223.

59. Silveira, C.R.F., Corveloni, A.C., Caruso, S.R., Macêdo, N.A., Brussolo, N.M., Haddad, F., Fernandes, T.R., de Andrade, P.V., Orellana, M.D., and Guerino-Cunha, R.L. (2022). Cytokines as an important player in the context of CAR-T cell therapy for cancer: Their role in tumor immunomodulation, manufacture, and clinical implications. Front. Immunol. *13*, 947648.

60. Zhou, J., Jin, L., Wang, F., Zhang, Y., Liu, B., and Zhao, T. (2019). Chimeric antigen receptor T (CAR-T) cells expanded with IL-7/IL-15 mediate superior antitumor effects. Protein Cell *10*, 764–769.

61. Du, L., Nai, Y., Shen, M., Li, T., Huang, J., Han, X., Wang, W., Pang, D., and Jin, A. (2021). IL-21 Optimizes the CAR-T Cell Preparation Through Improving Lentivirus Mediated Transfection Efficiency of T Cells and Enhancing CAR-T Cell Cytotoxic Activities. Front. Mol. Biosci. *8*, 675179.

62. Pharma Specialists. GMP Requirements for Supplier Qualification. https://www.pharmaspecialists.com/2022/02/gmp-requirements-for-supplier.html#gsc.tab=0.

YMTHE, Volume *33*

Supplemental Information

Optimizing CAR-T treatment: A T^{2}EVOLVE guide to raw and starting material selection

Sergio Navarro, Carole Moukheiber, Susana Inogés Sancho, Marta Ruiz Guillén, Ascensión López-Díaz de Cerio, Carmen Sanges, Toshimitsu Tanaka, Sylvain Arnould, Javier Briones, Harry Dolstra, Michael Hudecek, Rashmi Choudhary, Inga Schapitz, Manel Juan, Nina Worel, Delphine Ammar, Maik Luu, Mirko Müller, Bernd Schroeder, Hélène Negre, and Paul Franz

Table S1: *Guidelines pertaining to the raw/starting materials used for CAR-T cell products manufacturing.*

Document type	Reference	Title	Topics	Note
		**General ATMP guidance with chapters on raw/starting materials**		
EMA guideline	EMA/CAT/80183/2014 (replacing CPMP/BWP/ 3088/99)	**Guideline on the quality, non-clinical and clinical aspects of gene therapy medicinal products.**	Quality, non-clinical & clinical of ATMPs	**General guideline** presenting requirements for **gene therapy medicinal products,** for **MAA.** Raw/starting materials addressed in chapter 4 "Quality documentation".
EMA guideline	EMA/CAT/GTWP/671639/ 2008 Rev. 1	**Guideline on quality, non-clinical and clinical aspects of medicinal products containing genetically modified cells.**	Quality, non-clinical & clinical of ATMPs	**General guideline** presenting requirements for **products containing genetically modified cells**, for **MAA**. Raw/starting materials addressed in chapter 4 "Quality documentation".
EMA *draft* guideline	EMA/CAT/123573/2024 (previous reference: EMA/CAT/852602/2018)	**Guideline on quality, non-clinical and clinical requirements for investigational advanced therapy medicinal products in clinical trials.**	Quality, non-clinical & clinical of ATMPs	Guideline presenting requirements for **ATMPs** used in **clinical trials**. Raw/starting materials addressed in chapter 4 "Quality documentation".<br>*Draft guideline not adopted yet.*
European Commission guideline	C(2017) 7694	**Guidelines on Good Manufacturing Practice specific to Advanced Therapy Medicinal Products.**	Quality & GMP of ATMPs	**Guideline** on **GMP** for **ATMPs.** Raw/starting materials addressed in Chapter 7 ("Starting and raw materials"), Chapter 6 ("Documentation") and Chapter 2 ("Risk-based approach").
		**Guidance dedicated to vectors**		
EMA reflection paper	CHMP/GTWP/587488/07	**Reflection paper on quality, non-clinical and clinical issues relating specifically to recombinant adeno-associated viral vectors.**	Quality, non-clinical & clinical topics	Reflection paper on the development of **recombinant AAV vectors**.
EMA guideline	CHMP/BWP/2458/03	**Guideline on development and manufacture of lentiviral vectors.**	Development & manufacture	Guideline dedicated to **lentiviral vectors.**
		**Guidance applicable to biotechnology products and some vectors**		
ICH guideline	ICH Q5A Revision 2	**Viral safety evaluation of biotechnology products derived from cell lines of human or animal origin.**	Viral safety of biotechnology products (also applicable to certain vectors)	General guideline on viral safety of biotechnology products derived from cell lines of human or animal origin.<br>Following R2 revision (adopted in Nov 2023), guideline scope extended to become applicable to **certain viral vectors**, i.e., **only to viral vectors that are amenable to viral clearance steps.**
EMA guideline	CPMP/BWP/268/95	**The design, contribution and interpretation of studies validating the inactivation and removal of viruses.**	Viral safety of biotechnology products (also applicable to certain vectors)	Viral safety guideline on **the viral clearance studies (virus validation studies).** Guideline designed for biotechnology products, but **applicable to certain vectors (i.e., only to the vectors amenable to viral clearance steps).**

Guidance pertaining to materials of biological origin used to manufacture ATMPs				
European Pharmacopeia general chapter	Ph. Eur. 5.2.12.	**Raw materials of biological origin for the production of cell-based and gene therapy medicinal products.**	Quality of biological raw materials	Ph. Eur. chapter dedicated to **raw materials of biological origin** used for the production of **cell and gene therapy medicinal products.**
European Pharmacopeia general chapter	Ph. Eur. 5.1.7.	**Viral safety.**	Viral safety of biological materials	Ph. Eur. chapter dedicated to the **viral safety**, applicable to **materials of biological origin** used for the production of **cell and gene therapy medicinal products.**
European Pharmacopeia general chapter	Ph. Eur. 5.2.8.	**Minimising the risk of transmitting animal spongiform encephalopathy agents via human and veterinary medicinal products.**	Viral safety of biological materials	Ph. Eur. chapter dedicated to the **prion risk**, applicable to **materials of biological origin** used for the production of **ATMPs.**
European Pharmacopeia monograph	Ph. Eur. Monograph 2262	**Bovine serum monograph.**	Viral safety of biological materials	Ph. Eur. monograph dedicated to **bovine serum**.<br>Some parts of the monograph (such as viral testing) applicable to **bovine serum used as raw material** for the production of **ATMPs.**
European Pharmacopeia monograph	Ph. Eur. Monograph 0853	**Human plasma for fractionation monograph.**	Viral safety of biological materials	Ph. Eur. monograph dedicated to **human plasma** for fractionation. Some parts of the monograph (such as viral testing) applicable to **human serum derived products used as raw material** for the production of **ATMPs.**
EMA (CHMP/CAT) position paper	EMA/CHMP/BWP/353632 /2010	**CHMP/CAT position statement on Creutzfeldt-Jakob disease and advanced therapy medicinal products.**	Viral safety of biological materials	Position paper on the **prion risk** for **allogenic ATMPs**.<br>Applicable to **materials of biological origin** used for the production of **ATMPs.**
EMA guideline	EMA/CHMP/BWP/706271 /2010 (replacing CPMP/BWP/269/95)	**Note for guidance on plasma derived medicinal products.**	Viral safety of biological materials	Guideline dedicated to plasma-derived medicinal products. Principles for **collection of plasma-derived starting material** applicable to **allogenic leukapheresis** (used to manufacture **allogenic ATMPs**).
EMA guideline	EMEA/410/01	**Note for guidance on minimising the risk of transmitting animal spongiform encephalopathy agents via human and veterinary medicinal products.**	Viral safety of biological materials	Guideline applicable to **all ruminant-derived materials** used to manufacture medicinal products, including ATMPs.
EMA guideline	EMEA/22314/02	**Position Paper on re-establishment of working seeds and working cell banks using TSE compliant materials.**	Viral safety of biological materials	Position paper on the use of **ruminant-derived materials used for cell banks preparation.** Applicable to **recombinant vectors cell banks.**
EMA guideline	EMA/CHMP/BWP/457920 /2012 Rev 1 (replacing CPMP/BWP/1793/02)	**Guideline on the use of bovine serum in the manufacture of human biological medicinal products.**	Viral safety of biological materials	Guideline applicable to **all materials derived from bovine serum**, used to manufacture medicinal products.
EMA guideline	EMA/CHMP/BWP/814397 / 2011	**Guideline on the use of porcine trypsin used in the manufacture of human biological medicinal products.**	Viral safety of biological materials	Guideline applicable **to all materials derived from porcine trypsin**, used to manufacture medicinal products.

Table S2: *Different culture media used for CAR-T cell manufacturing.*

Type of Media	Name	Supplier
Serum-dependent media	RPMI-1640	Thermo Fisher Scientific
	AIM-V	Thermo Fisher Scientific
	CTS OpTimizer	Thermo Fisher Scientific
	IMDM	Thermo Fisher Scientific
Serum-free media or defined culture media	DMEM	Thermo Fisher Scientific
	mTESR	Stem Cell Technologies
	StemLine T cell expansion	SIGMA
	Prime XV cell expansion XSFM	Irvine Scientific
	Stem XV serum free human T cell base medium	R and D
	X-VIVO10	Lonza
	TexMACS	Miltenyi Biotec
	CellGenix T cell Medium	Cellgenix
	Prime-XV T cell CDM	Fujifilm
	LymphoONE T cell expansion Xenon-free Medium	Takara

Table S3: *Information required for a questionnaire for the selection of a raw material supplier.*

Area	Information requirements
**General Information**	- Contact details, number of staff, turnover, and organizational structure, as well as manufacturing sites (via Site Master File - SMF).<br>- It is recommended that a quality agreement or contract be established to clearly divide responsibilities between all parties involved.<br>- Access should be requested for auditing purposes to facilities, personnel, documents and pertinent financial information.<br>- Provision of information regarding the Quality Management System and compliance with regulatory requirements, including certification or accreditation level according to the ISO9000 family norm, GMP, or GLP is crucial.<br>- The availability of a Quality Manual or (Master) SOP for quality policy should be requested.
**Quality Assurance (QA) and Quality Control (QC)**	- Respectively, responsible personnel like Quality Managers.<br>- Questions about the system for Change Control and the management of Deviations.<br>- Data regarding inspections by authorities and/or other notified bodies if applicable.<br>- Information pertaining to Process Design and monitoring of problems, as well as the system for Corrective Actions and Preventive Actions (CAPA).
**Product and Process Innovation**	- Product and/or Process Innovation activity and provision of design improvements or innovations.
**Incoming materials**	- Tracing of incoming materials by lot or part number to the product, so the Chain of Identity (CoI) is guaranteed.<br>- Business continuity plans (or equivalent) that describe emergency measures taken in case of unexpected events.<br>- Provision of Certificate of Analysis (CoA) of all products.
**Quality Management Reports (QMR)**	- Concepts to drive continuous improvements and Quality Management Reports (QMR).

Table S4: *Additional information to consider before selecting a raw material supplier.*

**Expertise**	Current and future capabilities by scaling up to industrialization.
**Timing**	Subcontracting aspects, such as external QC, storage, and shipment.
**Stability**	The stability of the contract development and manufacturing organization (CDMO), including long-term viability and sustainability.
**Technology Transfer (TT)**	Possibility of transitioning to another CMO/provider due to mergers, acquisitions, or inability to scale up in terms of quantity and quality.
**Turnover of teams**	Ensuring continuity and expertise.
**Intellectual Property (IP)**	Protection and confidentiality measures.
**Cell bank** *if applicable*	Storage and maintenance.
**Costs**	Research and Development (R&D) compared to Good Manufacturing Practice (GMP) grade and considerations for change management.
**Devices used**	Equipment and technology employed in the manufacturing process.
